# Measuring 3D orientation of nanocrystals via polarized luminescence of rare-earth dopants

**DOI:** 10.1038/s41467-021-22158-4

**Published:** 2021-03-29

**Authors:** Jeongmo Kim, Reinaldo Chacón, Zijun Wang, Eric Larquet, Khalid Lahlil, Aymeric Leray, Gérard Colas-des-Francs, Jongwook Kim, Thierry Gacoin

**Affiliations:** 1 Laboratoire de Physique de la Matière Condensée, CNRS, École Polytechnique, Institut Polytechnique de Paris, Palaiseau, France; 2 Laboratoire Interdisciplinaire Carnot de Bourgogne (ICB), UMR 6303 CNRS, Université Bourgogne Franche-Comté, 9 Avenue Savary, BP 47870, Dijon, cedex France

**Keywords:** Imaging techniques, Imaging and sensing

## Abstract

Orientation of nanoscale objects can be measured by examining the polarized emission of optical probes. To retrieve a three-dimensional (3D) orientation, it has been essential to observe the probe (a dipole) along multiple viewing angles and scan with a rotating analyzer. However, this method requires a sophisticated optical setup and is subject to various external sources of error. Here, we present a fundamentally different approach employing coupled multiple emission dipoles that are inherent in lanthanide-doped phosphors. Simultaneous observation of different dipoles and comparison of their relative intensities allow to determine the 3D orientation from a single viewing angle. Moreover, the distinct natures of electric and magnetic dipoles originating in lanthanide luminescence enable an instant orientation analysis with a single-shot emission spectrum. We demonstrate a straightforward orientation analysis of Eu^3+^-doped NaYF_4_ nanocrystals using a conventional fluorescence microscope. Direct imaging of the rod-shaped nanocrystals proved the high accuracy of the measurement. This methodology would provide insights into the mechanical behaviors of various nano- and biomolecular systems.

## Abstract (web summary)

Determining the orientation of nanoscale objects in three-dimensional space has typically required complicated optical setups. Here, the authors develop a simple method to retrieve the 3D orientation of luminescent, lanthanide-doped nanorods from a single-shot emission spectrum.

## Introduction

Luminescent molecules or nanoparticles are universally used as labels to observe small objects such as bio-macromolecules. The position and movement of the target object can be precisely detected and tracked by virtue of the bright light emission of the point labels. Current super-resolution fluorescence microscopy techniques achieve a nanometer-scale spatial resolution far beyond the light diffraction limit^1,2^, and the advanced labels can even signal chemical and biomolecular events by FRET (Förster resonance energy transfer)^3,4^ or SPR (surface plasmon resonance) effects^5–7^. However, for most microscopic systems of interest, their rotational motions remain largely unexplored while this mechanical degree of freedom is often the key to understand the functionality of the system (e.g., molecular motors^8–10^, protein folding^11^, assembly of anisotropic objects^12,13^). Tracking the rotation of a small object with the size below the wavelength of light is a challenging problem independent of tracking the translation. It requires discerning the spatial orientation of a point object without visible shape. Thus, the emitted light must contain the information about its orientation that can be quantitatively analyzed by the observer.

A principal way to realize this is to examine the polarization of an anisotropic emission distribution in space, which lies in the nature of vector field of optical transition dipoles in luminescent species^14,15^. The dipole axis parallel to the polarization is determined by the intrinsic nature of the emitter particle, molecule, or cluster, which can be rigidly tagged with the target object. The orientation of the target can be deduced by taking into account the measured polarization. In this principle, molecular fluorescent dyes^8–10,16,17^, quantum rods^18–22^, and nanocrystals exhibiting second-harmonic generation (SHG)^23–27^ or upconversion emission^28,29^ have been used as probes to monitor the rotational dynamics of various nano-objects. A detailed comparison of the existing methods is shown in Supplementary Table 1.

**Fig. 1:**
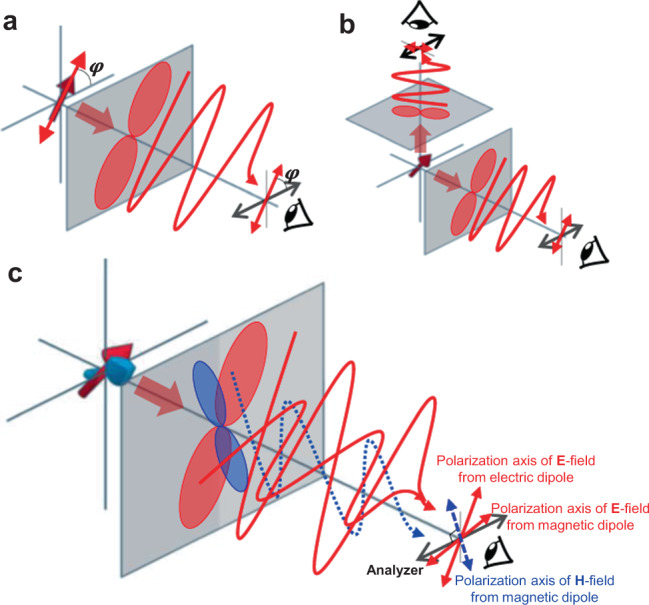
Schematic illustrations of the emission dipoles and their polar diagrams. The 3D arrows indicate the emission dipoles and the dumbbells indicate the polar diagrams: the measurable field intensity as a function of the angle of analyzer on the observation planes (grey plane). The wavy lines depict the oscillating electric field (red) and magnetic field (blue) polarized along the dipole axis. **a** A single electric dipole is oriented parallel to the observation plane. The dipole’s in-plane orientation angle can be determined from one polar diagram. **b** A single electric dipole with out-of-plane orientation. The projections of the dipole on two different observation planes render two polar diagrams, from which the three-dimensional (3D) dipole orientation can be determined. **c** Two dipoles with electric (red arrow) and magnetic (blue arrow) dipolar natures are oriented out-of-plane while they are coupled with an arbitrary fixed angle with respect to each other. Each dipole renders a distinct polar diagram (blue dumbbell — magnetic polar diagram/red dumbbell — electric polar diagram) on the same observation plane, from which the 3D orientation of the coupled dipoles can be determined.

Such a polarimetry is simple when observing the in-plane (2D) orientation of a probe determined by one variable *φ*, the angle between the dipole axis and the analyzer (polarizer in front of the detector). The measured intensity of the polarized emission versus *φ* follows a sinusoidal function, illustrated as a dumbbell-shaped polar diagram in Fig. 1a. The *φ* value can be determined from the observed polar diagram. However, when regarding the orientation in 3D space, two spherical angles (*θ*-polar and *φ*-azimuthal) contribute together to the polarized emission intensity measured from the observer. In order to determine these two angles (*θ*, *φ*), it is necessary to measure the emission polarization from at least two different viewing angles. Each measurement projects the dipole on the observation plane, so the orientations of the two polar diagrams allow to reconstruct *θ* and *φ* (Fig. 1b). Otherwise, two unparallel excitation beams can be used while observing through one viewing angle^14^. These, namely, stereoscopic methods have been used for studying the rotational dynamics of several systems (e.g., walking motion of myosin V^19,30^, rotational diffusion of rod-like particle^31–33^), but they require a rather complicated optical instrumentation with separate beam paths and multiple detectors and/or excitation sources, which is incompatible with conventional microscopes. Moreover, the accuracy of the orientation analysis can easily deteriorate because the measured signal intensity can vary not only with the dipole orientation but also with many other intrinsic and extrinsic sources such as photo-bleaching^34,35^ or -blinking^36–38^, polydispersity of the emitters^39^, experimental errors from the sensitive polarizing optics, and instrumental noise. An alternative approach, fluorescence correlation spectroscopy, monitors the intensity fluctuation of the polarized emission from which the rotational diffusion coefficient is obtained. It is efficient to study the time scale of rotational dynamics but cannot measure the absolute values of *θ* and *φ*^31–33^.

The recent approaches using a microscope objective with large numerical aperture (NA) have reduced such uncertainties by analyzing the integrative signal from continuous viewing angles over the large NA. Back-focal plane/defocusing imaging^40–44^ in this principle let the photons emitted along different directions fall on different regions of the camera detector making a specific image pattern, from which the dipole orientation can be deduced. A single-shot image pattern can provide the 3D orientation in this way. However, the numerical modeling for the pattern analysis is heavy and sensitive to the optical parameters (e.g., refractive index, focal depth, aberration)^18,45^, which remains as a limitation. An alternative method without pattern analysis while measuring at one viewing angle is to use an emitter composed of degenerate 2D dipoles in quantum dots^18,22^. In this case, measuring the 3D orientation requires a precise scanning of the emission intensity profile with non-zero minimum while rotating the analyzer in many steps. This method is thus not suitable when observing unstable or dynamically rotating systems, and quantum dots generally have a polydispersity issue^39^. Therefore, it is still challenging to establish a straightforward method to instantly and accurately measure the orientation of microscopic emitters.

Here, we present a methodology of 3D orientation analysis based on an instant measurement without a model-dependent calculation while achieving unprecedented angular accuracy and simple optical setup compatible with conventional microscopes. Instead of the stereoscopic analysis of a single emission dipole, we employ lanthanide luminophores exhibiting multiple emission dipoles with different natures of polarization — electric dipoles (ED) and magnetic dipoles (MD) — and spectroscopically analyze them from a single viewing angle.

Lanthanides exhibit multiple transition dipoles with separate emission wavelengths that can be observed simultaneously using a spectrometer. The mutually different polarization behaviors of ED and MD deliver the information on the 3D orientation through a single viewing angle (Fig. 1c). This feature is manifested as a varying line shape of the photoluminescence spectrum as a function of the angle of analyzer. The orientation analysis can thus be treated as a simple ratiometric analysis of the spectrally resolved emission peaks. Only a single spectrogram is sufficient to determine *θ* and *φ* because ED and MD offer two simultaneous equations (See Results section). Although the global intensity of the luminescence may fluctuate due to the above mentioned causes, the peak ratiometry depends exclusively on the orientation of the luminophore guaranteeing the accuracy of the calculated *θ* and *φ*. Furthermore, this method enables to measure collective partial orientation of many emitters together (e.g., self-assembly). In this case, the three variables — orientation of the director (*θ*, *φ*) and the order parameter (*f*) — can be determined with only two spectrograms observed with different analyzer angles: four simultaneous equations can be set with two orthogonal polarizations and ED-MD natures^46^.

When lanthanide ions are doped in a crystalline host, each energy level in 4f^n^ configuration is further split into several sublevels by the crystal field^47^. Each sublevel transition appears as a narrow emission peak related to a specific transition dipole. The dipole orientation with respect to the crystal axis is distinct for each sublevel and depends exclusively on the local site symmetry of the lanthanide dopant^48^. This provides a crucial advantage that the spectral profile of the polarized emission is not influenced by the size and morphology of nanocrystals^48^. Hence, the polarized emission spectra obtained from a sample with a known orientation are valid as a reference for other particles with the same crystal structure. This is not the case for other types of emitters such as quantum dots whose emission polarization sensitively varies with the particle size and shape^39^.

In this study, as a proof of concept of this method, we investigated the 3D orientation of monocrystalline sodium yttrium fluoride (NaYF_4_) nanorods doped with trivalent europium (Eu^3+^) ions. NaYF_4_ is an efficient crystalline matrix for lanthanide doping and is widely studied for applications in upconversion probes^49–52^ and nanothermometry^53,54^. As compared to other common emitting ions, Eu^3+^ ion shows sharp and well-defined emission peaks, thanks to the non-degenerate upper level of its main emission (^5^D_0_ → ^7^F_J_), which is ideal for the spectroscopic orientation analysis. Nanocrystals of NaYF_4_:Eu with a micrometer length were synthesized in order to compare the orientation directly seen by the optical microscope with the spectroscopically calculated value. We considered an optically homogeneous environment where NaYF_4_:Eu nanorods are embedded with random orientations in a thick poly vinyl alcohol (PVA) film deposited onto a glass substrate.

## Results

### Polarized photoluminescence of Eu^3+^ doped NaYF_4_ nanocrystal

**Fig. 2:**
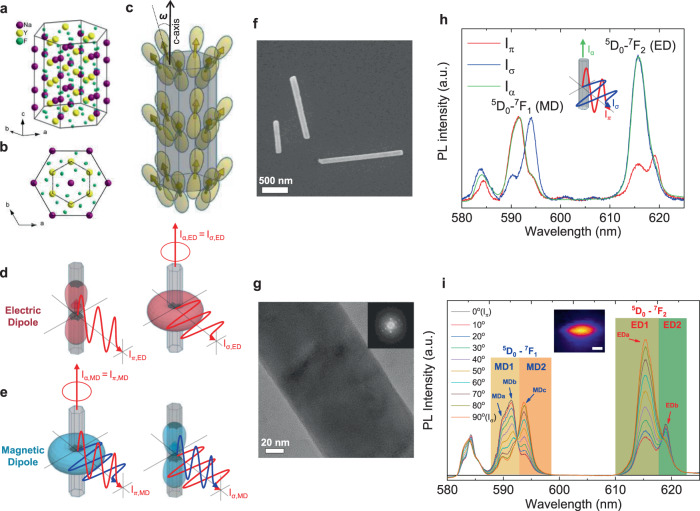
Dipole configurations and polarized spectra of the emission of Eu^3+^ doped in NaYF_4_ nanocrystal. **a**, **b** Illustrations of the hexagonal NaYF_4_ crystallographic structure (**a**) in 3D perspective view and (**b**) projected on the basal plane. The Y^3+^ sites are substituted when doped with Eu^3+^. **c** Schematic illustration of one kind of emission dipoles at the Eu^3+^ sites oriented with a certain polar angle (*ω*) with respect to the c-axis. The site symmetry determines *ω*, and the crystal symmetry induces the hexagonal arrangement of the dipoles. **d**, **e** Schematic illustrations of the symmetric ensemble of *ω*-angled dipoles decomposed to two orthogonal components: a 1D dipole parallel to the c-axis and a 2D dipole parallel to the basal plane. **d** For electric dipole (ED), the 1D dipole contributes to the π-polarization and the 2D dipole contributes to the σ-polarization equal to the α-configuration. **e** This relation is inversed for magnetic dipole (MD) as its 1D dipole contributes to the σ-polarization and its 2D dipole contributes to the π-polarization equal to the α-configuration. **f** Scanning electron microscopy (SEM) image and (**g**) high-resolution transmission electron microscopy (HR-TEM) image with fast Fourier transformation (inset) of NaYF_4_:Eu nanorods. **h** Polarized photoluminescence (PL) spectra in π, σ, and α configurations of NaYF_4_:Eu nanorods under the excitation at 394 nm (^7^F_0_ → ^5^L_6_). These spectra were obtained from a colloidal solution in which the nanorods were uniformly aligned by external electric field (Supplementary Fig. 4). **i** Polarized PL spectra of a single NaYF_4_:Eu nanorod lying on a plane substrate with varied analyzer angles. The blue and red arrows indicate the three main peaks (MDa–c) in the ^5^D_0_ → ^7^F_1_ transition and the two main peaks (EDa–b) in the ^5^D_0_ → ^7^F_2_ transition. The selected wavelength ranges (MD1, MD2, ED1, and ED2) to perform the area under curve ratiometry are highlighted in different colors. The inset is an image of the nanorod obtained by scanning the PL signal using a piezo-stage (scale bar: 1 µm). The full emission spectrum of NaYF_4_:Eu is shown in Supplementary Fig. 5.

The crystallographic structure of NaYF_4_ in hexagonal phase is illustrated in Fig. 2a, b. The projection on the basal plane normal to the c-axis (Fig. 2b) shows the hexagonal symmetry of Y^3+^ sites in the lattice, which can be substituted by Ln^3+^ dopants. The nanocrystal synthesis using a stoichiometric mixture of Y^3+^ and Eu^3+^ precursors leads to a homogeneous and random occupation of Y^3+^ sites by Eu^3+^ with a well-controlled doping percentage^55,56^. One transition dipole of Eu^3+^ is assumed to have a certain polar angle (*ω*) with respect to the c-axis of the crystal that is parallel to the geometrical axis of the nanorod (Fig. 2c). The ensemble of the dipoles in different sites performs a hexagonal symmetry on average, which can be decomposed as an axial component and a radial component (parallel and perpendicular to the c-axis respectively) (Fig. 2d, e). The axial component has a 1D nature (dumbbell shape) identical to a single dipole whereas the radial component has a degenerate 2D nature (horn torus shape) due to the in-plane distribution of individual dipoles. The symmetry and group theory predicts either purely axial or purely radial dipole for sublevel emission for the C_3h_ symmetry group of the Y site^57–59^. However, symmetry breaking occurs to a lower C_s_ site symmetry because of the larger ionic radius of Eu^3+^ in comparison to Y^3+^^60^. Most of the few reported experimental observations including NaYF_4_:Eu of our case show superposition of the two components that is manifested as varied degrees of polarization for different sublevel peaks^61–63^. We ascribe this to the tilting of dipoles (0° < *ω* < 90°) as schematized in Fig. 2c^60,64,65^.

In case of ED (Fig. 2d), where the electric field oscillates along the dipole axis, the light radiated from the axial component is polarized parallel (π) to the c-axis and the light from the radial component is polarized perpendicular (σ) to the c-axis. In case of MD (Fig. 2e), by contrast, as the electric field is perpendicular to the magnetic field oscillating along the dipole, the light from the axial and radial components are σ- and π-polarized respectively. For both ED and MD, the light propagating along the c-axis (α) is unpolarized as it originates from the 2D nature of the radial components (the unpolarized feature of the α-configuration is shown in Supplementary Fig.  1).

The SEM image in Fig. 2f shows a straight rod-like morphology of the NaYF_4_:Eu nanocrystals used in this study. Their sizes are quite polydisperse (length = 1184 ± 430 nm, width = 117 ± 24 nm) which is a typical feature of solvothermally synthesized nanorods (the size distribution histogram is presented in Supplementary Fig. 2). The high-resolution TEM and XRD analyses (Fig. 2g, Supplementary Fig. 3) confirmed their hexagonal single crystalline nature.

Figure 2h shows the polarized photoluminescence emission spectra of these NaYF_4_:Eu nanorods in three main configurations (π, σ, and α). These spectra were obtained from aligned colloidal nanorods under externally applied electric field following the method described in our previous work^66,67^. (the experimental detail is also shown in Supplementary Fig. 4). The intense group of peaks at 587–597 nm corresponds to the ^5^D_0_ → ^7^F_1_ transition of Eu^3+^ which originates from MDs, and the one at 610–622 nm corresponds to the ^5^D_0_ → ^7^F_2_ transition from EDs. Each narrow peak (partially overlapping with the adjacent peaks) corresponds to one sublevel transition split by the crystal field^47^. One can see that the π and σ spectra are dramatically different to each other and the α spectrum is identical to the π spectrum for the MD transition (^5^D_0_ → ^7^F_1_) and to the σ spectrum for the ED transition (^5^D_0_ → ^7^F_2_). This unique polarization behavior confirms the above-described axial/radial and ED/MD dipolar nature (Fig. 2d, e). Consequently, π, σ, and α spectra are all different to each other considering the whole spectral range, and a spectrum observed from an arbitrary viewing angle would be a weighted sum of the three reference spectra depending on the viewing angle (i.e., rod orientation with respect to the observer).

An isolated single NaYF_4_:Eu nanorod shows the same π and σ spectra and the continuous line shape variation between them when the analyzer was rotated every 10° with respect to the rod axis lying on a plane substrate (Fig. 2i). We have previously demonstrated a similar aspect of the polarized emission of Eu^3+^ doped in LaPO_4_ nanocrystals^46,64^. The degree of polarization (DOP) for each peak is defined as**:**

1 $${\mathrm{DOP}} = (I_\pi - I_\sigma )/(I_\pi + I_\sigma )$$

where I_π_ and I_σ_ stand for the relative intensities of π and σ configurations. The three main peaks within ^5^D_0_ → ^7^F_1_ transition (MDa: 589.9 nm, MDb: 591.4 nm, and MDc: 593.7 nm) have DOPs of 0.53, 0.56, and −0.56 respectively, and the two main peaks in ^5^D_0_ → ^7^F_2_ transition (EDa: 615.3 nm, and EDb: 619.0 nm) have DOPs of −0.56 and 0.21. Therefore, in both MD and ED transitions, there exist at least two peaks with high contrast of DOPs, which ensures a precise ratiometric analysis. The well-defined three peaks in the ^5^D_0_ → ^7^F_1_ transition confirm that ^7^F_1_ level is split into three sublevels by the crystal field in a low symmetry (C_s_) of Eu^3+^ sites^68^. As the low symmetry would split the ^5^D_0_ → ^7^F_2_ level into five sublevels^68^, the broad EDa peak seems to be indeed several sublevels that are merged together. When regarding, for simplicity, each of EDa and EDb as one dipole, the positive DOP of EDa (I_π_ > I_σ_) indicates the dipole axis close to the c-axis (*ω* < 45°) and the negative DOP of EDb indicates the dipole lying close to the basal plane (*ω* > 45°). This relation is inversed for MD peaks due to the orthogonality of electric and magnetic fields (i.e., positive DOP of MD indicates its dipole axis close to the basal plane).

In the next sections, the analytical method is provided to determine the 3D orientation of a nanorod based on the intrinsic emission polarization property of NaYF_4_:Eu described above.

### Orientation analysis methodology

**Fig. 3:**
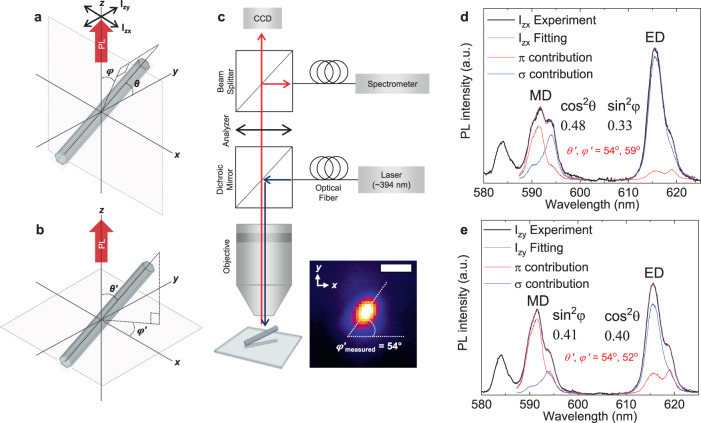
Orientation analysis of a single NaYF_4_:Eu nanorod by spectral fitting. **a**–**b** Schematic illustration of a hexagonal crystalline NaYF_4_:Eu nanorod with an arbitrary 3D orientation denoted by the spherical angles (*θ*, *φ*) or (*θ*′, *φ*′) when the polar axis is (**a**) *y*-axis or (**b**) *z*-axis respectively. The observation plane is fixed as xy-plane. **c** A scheme of the optical microscopy setup used to conduct the orientation analysis. The inset is a photograph of the photoluminescence (PL) of a single nanorod captured by the CCD camera (scale bar: 1 µm). The in-plane angle (*φ*′) of the nanorod was found as the long axis of the ellipsoidal shape of this image. **d**, **e** Spectral fitting analysis using a polarized PL spectrum I_zx_ or I_zy_ obtained with the analyzer (**d**) parallel to the *x*-axis or (**e**) parallel to the *y*-axis. Each analysis considers both ED and MD transitions in order to determine *θ* and *φ* from a single spectrum using Eqs. 4–9. The calculated values of the trigonometric functions of (*θ*, *φ*) and the absolute values of (*θ*′, *φ*′) are presented in the figure. The results of the same analysis for a large number of particles are shown in the supplementary information (Supplementary Fig. 8, Supplementary Data 1).

For a nanorod with an arbitrary orientation in the laboratory frame by polar and azimuthal angles (*θ*, *φ*) (Fig. 3a), the emission intensities collected at two orthogonal polarization angles, I_zx_ and I_zy_ (first and second subscripts refer to the axes of propagation and polarization, respectively), have contributions of the π, σ, and α configurations as their projections on the measurement axis (see Supplementary Fig. 6 for graphic explanation). For a given wavelength (λ):

2 $${\mathrm{I}}_{{\mathrm{zx}}}\left( \lambda \right) = {\mathrm{I}}_\pi \left( \lambda \right) \cdot {\mathrm{sin}}^2\theta \cdot {\mathrm{sin}}^2\varphi + {\mathrm{I}}_\sigma \left( \lambda \right) \cdot {\mathrm{cos}}^2\theta + {\mathrm{I}}_\alpha \left( \lambda \right) \cdot {\mathrm{sin}}^2\theta \cdot {\mathrm{cos}}^2\varphi$$

3 $${\mathrm{I}}_{{\mathrm{zy}}}\left( \lambda \right) = {\mathrm{I}}_\pi \left( \lambda \right) \cdot {\mathrm{cos}}^2\theta + {\mathrm{I}}_\sigma \left( \lambda \right) \cdot {\mathrm{sin}}^2\theta \cdot {\mathrm{sin}}^2\varphi + {\mathrm{I}}_\alpha \left( \lambda \right) \cdot {\mathrm{sin}}^2\theta \cdot {\mathrm{cos}}^2\varphi$$

where I_π_, I_σ_, and I_α_ indicate the relative intensities of the π, σ, and α configurations.

As the α spectrum is identical to the σ spectrum for ED and to the π spectrum for MD (Fig. 2h), the above equations can be rewritten separately as functions of only I_π_ and I_σ_ as:

for ED transition,

4 $${\mathrm{I}}_{{\mathrm{zx}},{\mathrm{ED}}}\left( \lambda \right) = {\mathrm{I}}_{\pi ,{\mathrm{ED}}}\left( \lambda \right) \cdot {\mathrm{sin}}^2\theta \cdot {\mathrm{sin}}^2\varphi + {\mathrm{I}}_{\sigma ,{\mathrm{ED}}}\left( \lambda \right) \cdot ({\mathrm{cos}}^2\theta \cdot {\mathrm{sin}}^2\varphi + {\mathrm{cos}}^2\varphi )$$

5 $${\mathrm{I}}_{{\mathrm{zy}},{\mathrm{ED}}}\left( \lambda \right) = {\mathrm{I}}_{\pi ,{\mathrm{ED}}}\left( \lambda \right) \cdot {\mathrm{cos}}^2\theta + {\mathrm{I}}_{\sigma ,{\mathrm{ED}}}\left( \lambda \right) \cdot {\mathrm{sin}}^2\theta$$

and for MD transition,

6 $${\mathrm{I}}_{{\mathrm{zx}},{\mathrm{MD}}}\left( \lambda \right) = {\mathrm{I}}_{\sigma ,{\mathrm{MD}}}\left( \lambda \right) \cdot {\mathrm{cos}}^2\theta + {\mathrm{I}}_{\pi ,{\mathrm{MD}}}\left( \lambda \right) \cdot {\mathrm{sin}}^2\theta$$

7 $${\mathrm{I}}_{{\mathrm{zy}},{\mathrm{MD}}}\left( \lambda \right) = {\mathrm{I}}_{\sigma ,{\mathrm{MD}}}\left( \lambda \right) \cdot {\mathrm{sin}}^2\theta \cdot {\mathrm{sin}}^2\varphi + {\mathrm{I}}_{\pi ,{\mathrm{MD}}}\left( \lambda \right) \cdot ({\mathrm{cos}}^2\theta \cdot {\mathrm{sin}}^2\varphi + {\mathrm{cos}}^2\varphi )$$

Hence, one can choose a single polarization axis (for either I_zx_ or I_zy_) to get two simultaneous equations on ED and MD from which the unknown *θ* and *φ* values can be calculated. As the ED and MD transitions occur simultaneously in lanthanides, a single emission spectrum is sufficient to determine the 3D orientation (*θ*, *φ*) of a nanorod. It is apparently possible also to choose one transition (either ED or MD) at two polarization axes (see Supplementary Fig. 7,8 and Supplementary Data 1). The choice of two equations among the four above can indeed be made as preferred considering the material property such as the DOP of the transition and the experimental condition such as type, number, and spectral resolution of the detectors.

The *y*-axis in the laboratory frame was chosen as the polar axis related to the (*θ*, *φ*) angles (Fig. 3a), which simplifies the Eqs. 4–7. A transformation of (*θ*, *φ*) into a new spherical coordinate with the *z*-axis as its polar axis (Fig. 3b) can be made by the equations:

8 $$\theta ^{\prime} = {\mathrm{cos}}^{ - 1}({\mathrm{sin}}\theta \cdot {\mathrm{cos}}\varphi )$$

9 $$\varphi ^{\prime} = {\mathrm{sin}}^{ - 1}\left( {{\mathrm{cos}}\theta {\mathrm{/}}\sqrt {1 - {\mathrm{sin}}^2\theta \cdot {\mathrm{cos}}^2\varphi } } \right)$$

where (*θ*′, *φ*′) are the polar and azimuthal angles in the new coordinate which is more intuitive for the observer: *φ*′ and *θ*′ correspond to the in-plane and out-of-plane orientation angles on the observation plane (see also Supplementary Eqs. 1–5). In order to determine the signs of (*θ*′, *φ*′), additional procedures are required as described at the end of the Results section.

### Optical measurement

In order to test the discussed methodology, we have prepared NaYF_4_:Eu nanorods that are embedded in a thick polymer film with random orientation. The individual dispersion of the nanorods was achieved by mixing citrate-functionalized nanorods with an aqueous solution of polyvinyl alcohol (PVA) at very low rod-concentration, which was then drop-casted on a glass coverslip. The refractive index of PVA is ~1.5, close to ~1.52 for standard glass coverslip. The optical environment surrounding the NaYF_4_:Eu nanorods can thus be regarded as homogeneous and not significantly distorting the emission dipoles even near the polymer–glass interface. Photoluminescence measurements and nanorod observations were carried out using a conventional optical microscope equipped with a fiber-coupled excitation laser (*λ*_ex_ = 394 nm, matching with the ^7^F_0_ → ^5^L_6_ transition line), a spectrometer, and a CCD camera (Fig. 3c).

A photograph of an emitting nanorod captured by the CCD camera (Fig. 3c-inset) shows a slightly elliptical shape of the spot due to the out-of-plane tilt of the embedded nanorod (length ~ 1 µm). The in-plane projection of the nanorod length in this case is close to the spatial resolution of the optical microscope. The long axis of the ellipsoid provides the azimuthal orientation *φ*′ of the nanorod. Its measured value of 54° will be compared below with the calculated one from the polarized emission spectra. Only the in-plane projection of the 3D orientation of the nanorod is barely discernible even though the rod size was controlled to be relatively large. Particles smaller than the spatial resolution of the microscope or in vertical orientation (*θ*′ ~ 0°) will appear in circular shape on such images.

### Orientation analysis by spectral fitting

The polarized photoluminescence emission spectra were collected using a rotating analyzer in front of the spectrometer (Fig. 3d, e). As discussed above, the line shape of each spectrum on both ED and MD ranges contains the information on the 3D orientation of the nanorod. By fitting one of these spectra (I_zx_ or I_zy_) separately in ED and MD wavelength ranges with the reference π- and σ-polarized spectra (I_π_, I_σ_), the (*θ*, *φ*) values were obtained using Eqs. 4–7, which were then converted to (*θ*′, *φ*′) using Eqs. 8–9. It can be seen in Fig. 3d, e that the fitting curves (magenta lines) as weighted sums of the I_π_, I_σ_ curves (red and blue lines) match well with the observed I_zx_ and I_zy_ spectra (black lines). The two sets of (cos^2^θ, sin^2^φ, and *θ*′, *φ*′) values calculated from the two polarizations (I_zx_, I_zy_) are presented in the same figure. The *φ*′ values calculated from I_zx_ and I_zy_ are 59° and 52° respectively, while the measurement on the CCD-captured image was 54°. The several degrees of error and discrepancy between different selections of the analyzer angle are ascribed to be due to the experimental errors such as the alignment of analyzer and the polarization-dependent fluctuation of the background offset on the spectrograms. Supplementary Fig. 9–11 shows the examples of experimental errors and their propagation into orientation analysis. We conducted the same orientation analysis repeatedly on 33 randomly oriented single nanorods in polymer film and the mean absolute error for *φ*′ was 4.6° (Supplementary Data 1). The out-of-plane angle (*θ*′) remains incomparable as CCD images a projection. The deviation in (*θ*′, *φ*′) values were less than 4° in average compared with the values calculated either using a different polarization (I_zx_ or I_zy_ using both MD and ED transition) or using a different dipole transition (MD or ED emission using both I_zx_ and I_zy_ polarizations) (Supplementary Data 1), confirming a wide applicability of the proposed method to various materials and experimental conditions. The same orientation analysis is also conducted on 21 different nanorods directly deposited on the substrate without polymer: *θ*′ = 90° (Supplementary Data 1). In this case, the mean absolute errors for *θ*′ and *φ*′ were 13.1° and 3.2° (Supplementary Data 1). We found that the mean errors for *θ*′ and *φ*′ can vary depending on their absolute values because they are calculated from sinusoidal curves. The errors are relatively large when *θ*′ or *φ*′ is close to 0° or to 90° (i.e., when the rods are oriented parallel or perpendicular to the analyzer or to the substrate). This is why the mean error for *θ*′ is larger than that of *φ*′ for the rods lying on the substrate (*θ*′ = 90°). At this certain orientation condition of the nanorod, a small experimental error in the spectral fitting may cause the analysis to fail. See Supplementary Fig. 10 for the simulated error profiles based on Eqs. 8–9.

### Orientation analysis by area under curve ratiometry

**Fig. 4:**
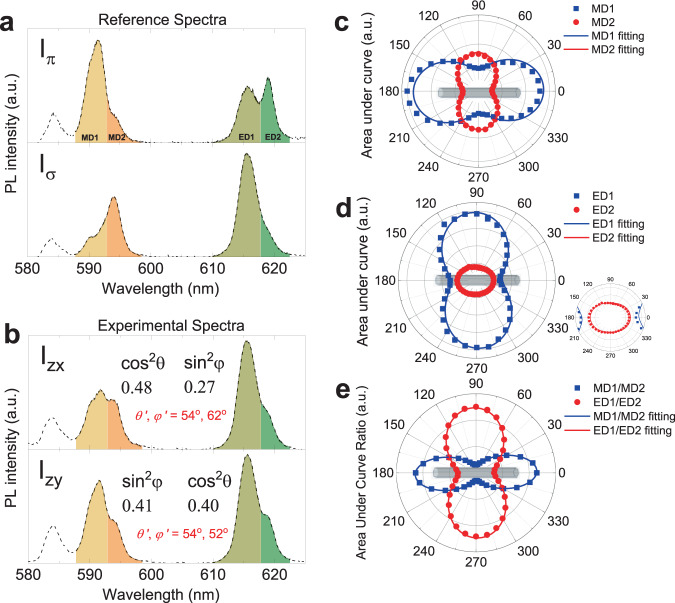
Orientation analysis of a single NaYF_4_:Eu nanorod by area under curve ratiometry (AUCR). **a** Reference emission spectra of the photoluminescence (PL) of a NaYF_4_:Eu nanorod polarized parallel (I_π_) and perpendicular (I_σ_) to the rod axis and (**b**) experimental spectra (I_zx_, I_zy_) of the nanorod with an orientation *θ*′, *φ*′ (also shown in Fig. 3d, e). The selected wavelength ranges (MD1, MD2, ED1, and ED2) used for AUCR are highlighted in different colors, from which the values of the trigonometric functions of (*θ*, *φ*) and the absolute values of (*θ*′, *φ*′) were calculated using Eqs. 8–13. The results of the same AUCR analysis for a large number of particles are shown in the Supplementary Data 1. **c**–**e** Polar diagrams and fitting curves of the selected areas under curve for (**c**) MD1 and MD2, (**d**) ED1 and ED2, and (**e**) their ratios MD1/MD2 and ED1/ED2.

The spectral fitting analysis minimizes the error on the calculation of (*θ*′, *φ*′) values. This requires spectra recorded with a spectrometer. Other types of detectors such as photomultiplier tube (PMT), avalanche photodiode (APD), or electron multiplying CCD are often preferred when higher photosensitivity or spatially resolved photography is desired. In such cases, the photons emitted in four different spectral ranges can be separately collected using a combination of dichroic mirrors and optical bandpass filters. The detected signal intensities are then proportional to the integrated areas under the spectrum curve in the selected ranges. The ratiometry between the signal intensities in different ranges can be used to calculate (*θ*′, *φ*′) angles instead of the spectral fitting analysis. The Eqs. 4–7, with the intensities as continuous functions of wavelength, can be rewritten with the integrated intensities for divided ranges referred to as ED1, ED2, MD1, and MD2 (Fig. 4a, b), from which the cos^2^θ and sin^2^φ can be expressed as: for ED transition,

10 $${\mathrm{cos}}^2\theta = \frac{{{\mathrm{k}}_{{\mathrm{zy}},{\mathrm{ED}}} \cdot {\mathrm{I}}_{\sigma ,{\mathrm{ED}}2} - {\mathrm{I}}_{\sigma,{\mathrm{ED}}1}}}{{{\mathrm{k}}_{{\mathrm{zy}},{\mathrm{ED}}} \cdot \left( {{\mathrm{I}}_{\sigma ,{\mathrm{ED}}2} - {\mathrm{I}}_{\pi ,{\mathrm{ED}}2}} \right) - \left({\mathrm{I}}_{\sigma ,{\mathrm{ED}}1} - {\mathrm{I}}_{\pi,{\mathrm{ED}}1}\right)}}$$

11 $${\mathrm{sin}}^2\varphi = \frac{{{\mathrm{k}}_{{\mathrm{zx}},{\mathrm{ED}}} \cdot {\mathrm{I}}_{\sigma ,{\mathrm{ED}}2} - {\mathrm{I}}_{\sigma ,{\mathrm{ED}}1}}}{{\left( {1 - {\mathrm{cos}}^2{\theta}} \right) \cdot \left[\left( {{\mathrm{I}}_{\pi ,{\mathrm{ED}}1} - {\mathrm{I}}_{\sigma ,{\mathrm{ED}}1}} \right) - {\mathrm{k}}_{{\mathrm{zx}},{\mathrm{ED}}} \cdot \left( {{\mathrm{I}}_{\pi ,{\mathrm{ED}}2} - {\mathrm{I}}_{\sigma ,{\mathrm{ED}}2}} \right)\right]}}$$

where I_π,EDn_ and I_σ,EDn_ (*n* = 1 or 2) indicate the relative area under curve of the π and σ spectra (Fig. 4a) and k_zy,ED_ and k_zx,ED_ stand for the measured intensity ratios I_zy,ED1_/I_zy,ED2_ and I_zx,ED1_/I_zx,ED2_, respectively (Fig. 4b).

And for MD transition,

12 $${\mathrm{cos}}^2\theta = \frac{{{\mathrm{k}}_{{\mathrm{zx}},{\mathrm{MD}}} \cdot {\mathrm{I}}_{\pi ,{\mathrm{MD}}2} - {\mathrm{I}}_{\pi ,{\mathrm{MD}}1}}}{{{\mathrm{k}}_{{\mathrm{zx}},{\mathrm{MD}}} \cdot \left( {{\mathrm{I}}_{\pi ,{\mathrm{MD}}2} - {\mathrm{I}}_{\sigma ,{\mathrm{MD}}2}} \right) - \left({\mathrm{I}}_{\pi ,{\mathrm{MD}}1} - {\mathrm{I}}_{\sigma ,{\mathrm{MD}}1}\right)}}$$

13 $${\mathrm{sin}}^2\varphi = \frac{{{\mathrm{k}}_{{\mathrm{zy}},{\mathrm{MD}}} \cdot {\mathrm{I}}_{\pi ,{\mathrm{MD}}2} - {\mathrm{I}}_{\pi ,{\mathrm{MD}}1}}}{{\left( {1 - {\mathrm{cos}}^2\theta } \right) \cdot \left[\left( {{\mathrm{I}}_{\sigma ,{\mathrm{MD}}1} - {\mathrm{I}}_{\pi ,{\mathrm{MD}}1}} \right) - {\mathrm{k}}_{{\mathrm{zy}},{\mathrm{MD}}} \cdot \left( {{\mathrm{I}}_{\sigma ,{\mathrm{MD}}2} - {\mathrm{I}}_{\pi ,{\mathrm{MD}}2}} \right)\right]}}$$

where I_π,MDn_ and I_σ,MDn_ (*n* = 1 or 2) indicate the relative area under curve of the π and σ spectra (Fig. 4a) and k_zx,MD_ and k_zy,MD_ stand for I_zx,MD1_/I_zx,MD2_ and I_zy,MD1_/I_zy,MD2_, respectively (Fig. 4b).

The orientation determination proceeds similar with the spectral fitting analysis. One can choose either one polarization axis (I_zx_ or I_zy_) or one transition (ED or MD) and obtain a set of two *k* values among *k*_zx,ED_, *k*_zx,MD_, *k*_zy,ED_, and *k*_zy,MD_, then calculate (*θ*, *φ*) values using a set of two equations among Eqs. 10–13.

This ratiometric analysis was performed on the same polarized photoluminescence spectra used for the fitting analysis in order to compare the accuracies of the two methods. The reference areas under curve (I_π,MD1_, I_π,MD2_, I_π,ED1_, I_π,ED2_ and I_σ,MD1_, I_σ,MD2_, I_σ,ED1_, I_σ,ED2_) were measured on the reference π and σ spectra (Fig. 4a). The areas under curve on the experimental spectra (I_zx,MD1_, I_zx,MD2_, I_zx,ED1_, I_zx,ED2_ and I_zy,MD1_, I_zy,MD2_, I_zy,ED1_, I_zy,ED2_) were measured for the same spectral ranges (Fig. 4b), from which the *k* values (*k*_zx,MD_, *k*_zx,ED_, *k*_zy,MD_, and *k*_zy,ED_) were obtained and the (*θ*, *φ*) and finally (*θ*′, *φ*′) values were calculated using Eqs. 10–13 and Eqs. 8–9. The deviation in (*θ*′, *φ*′) values for a number of tested nanorod samples were less than 2° in average compared with the values calculated by the spectral fitting analysis (Supplementary Data 1). The mean absolute error is also similar with that of the spectral fitting analysis. (Supplementary Data 1). The area under curve ratiometry is thus a good alternative method of the spectral fitting analysis almost maintaining the accuracy of orientation measurement. The simulated error profile based on the Eqs. 12–13 is also presented in the S.I. (Supplementary Fig. 11).

The best precision of the ratiometry can be achieved by properly selecting the wavelength ranges (ED1, ED2, MD1, and MD2) to have the highest DOP in each range. As highlighted in the diagram of the series of reference spectra for varied analyzer angles (Fig. 2i), the similarly polarized neighboring sublevel peaks were grouped in one range. For example, the MDa (589.9 nm) and MDb (591.4 nm) peaks were grouped as MD1 and the MDc (593.7 nm) peak was defined as MD2. Fig. 4c, d shows polar diagrams of the in-planed oriented nanorod plotted for the varied areas under curve as a function of the analyzer angle (*χ*). The data points should in principle fit with a simple trigonometric equation (Eq. 14) implying a sum of the projections of the π and σ components:

14 $${\mathrm{I}}_{\upchi} = {\mathrm{I}}_\pi \cdot {\mathrm{cos}}^2{\upchi} + {\mathrm{I}}_\sigma \cdot {\mathrm{sin}}^2{\upchi}$$

However, the experimental points are not perfectly matching with the fitting lines for the Eq. 14. This typical mismatch is caused by signal fluctuation during scanning over *χ*-angles or by the beam distortion along the optical setup. The points for *χ* = 120° in Fig. 4d markedly deviate from the fitting line presumably due to the fluctuation of the excitation source. The asymmetry of the polar diagram for MD1 in Fig. 4c (left arc is larger than the right) seems to be due to a slight distortion of the emission beam path when rotating the analyzer. Nevertheless, the ratiometric polar diagrams of the *k* values (Fig. 4e) consistently match the fitting curves by virtue of the simultaneous measurements of the emission peaks in different wavelength ranges. Such a high precision of the *k*-polar diagrams was preserved even when the overall emission intensity was largely varied during scanning *χ* from 0° to 360° (Supplementary Fig. 12). This feature represents the outstanding accuracy of our spectroscopic orientation analysis by both the spectral fitting and area under curve ratiometry methods. Moreover, the *k*-polar diagrams (Fig. 4e) are narrower in cervical region due to the combination of the DOPs for different wavelength ranges. The DOP values for MD1/MD2 and ED1/ED2 are 0.77 and −0.57 respectively (for NA = 0.6), higher than the DOPs of each peak. This also assures a higher accuracy to determine (*θ*′, *φ*′) values as compared to an analysis based on measuring the variation of an absolute intensity of a single emission peak. We checked that the DOPs of five randomly selected nanorods with different sizes are all the same (Supplementary Fig. 13, Supplementary Table 2), confirming that the lanthanide emission is independent of the particle morphology and that the orientation analysis is robust against polydisperse systems.

The measured DOPs decrease as the NA of the microscope objective increases because the emission towards a wider solid angle is averaged (Supplementary Fig. 14, Supplementary Table 3)^65^. There is a tradeoff between the spatial resolution — for which large NA is favored — and the accuracy of orientation analysis — for which high DOPs by small NA are favored. An optimal NA is to be selected considering the specific experimental requirements. Besides, DOP can also be affected by other environmental and experimental factors such as spectral efficiency of the analyzer, the optical properties of the medium surrounding the emitter and the doping concentration of rare-earth ions in the host crystal^69,70^. The reference spectra (I_π_, I_σ_) and the experimental spectra (I_zx_, I_zy_) should therefore be collected in the same condition for the correct orientation analysis.

### Discriminating mirror angles (−*θ*′, −*φ*′)

Because the above equations provide the values of trigonometric functions, the *θ*′ and *φ*′ are indistinguishable from −*θ*′ and −*φ*′ creating four-fold mirror orientations. The in-plane mirror angle (−*φ*′) can be discriminated by measuring a spectrum at one supplementary analyzer angle between *x*- and *y*-axis (for example, I_z45°_). The reference *k*-polar diagram can be plotted based on Fig. 4e at the different analyzer angles (k_zx_, k_zy_, and k_z45°_) as a function of *φ*′, which can serve to establish a boundary condition to distinguish *φ*′ and −*φ*′. A detailed procedure is described in the S.I. (Supplementary Fig. 14). Discriminating the mirror orientation of *θ*′ is also technically possible if a measurement can be done on another viewing angle adopting the previous stereoscopic method (e.g., by tilting the sample or using a large NA objective with a diaphragm at different positions).

## Discussion

We have demonstrated a straightforward spectroscopic method to measure the three-dimensional orientation of nanocrystalline probes. We show that the large measurement uncertainty of previous methods using a single dipole emitter can be eliminated via ratiometric analysis of multiple transition dipoles inherent in lanthanide phosphors. The spectral line shape (i.e., relative probabilities of different emission dipoles) of lanthanides doped in a crystal structure is independent of the particle size, shape, and surface chemistry that are major factors governing other types of nano-emitters, which is a great advantage to analyze the absolute angles of orientation with high accuracy.

We used NaYF_4_:Eu nanorod as a model system, but any probe material with different dopants, composition, and structure (e.g., β-NaYF_4_:Er^3+^,Yb^3+^ upconverting nanoparticles^71^) exhibiting simultaneous ED and MD transitions can be used to measure the 3D orientation from a single-shot spectrum obtained with a fixed analyzer. Organometallic lanthanide chelates are potential orientation probes that are smaller and compatible with biomolecular targets. The ED and MD transition levels of different lanthanide ions are summarized in Supplementary Table 4 with a discussion on their applicability. If only one type of dipoles is available (either ED or MD), one can still measure the 3D orientation from two spectra obtained with two different analyzer angles, which still benefits from the accuracy of ratiometry and opens a diversified choice for probes even beyond lanthanide doped species. Depending on the measurement conditions and specific target of study, the proposed principle of analysis can be easily adapted to conventional microscopy setups with various designs and detector types. The precision of the measurement can be further improved by using a host crystal structure providing higher degrees of polarization (DOPs)^64^.

Such an analytic performance combined with a large choice of emitter material and optical instrument is promising for studying the orientation and rotational dynamics of a wide variety of systems in biology^19,30,72^, physics^28,46,71,73^ and colloidal science^74,75^, to mention a few.

## Methods

### Preparation of NaYF_4_:Eu nanorods

NaYF_4_ nanorods with 5% Eu-doping were hydrothermally synthesized following the procedure demonstrated in the reference^55,76,77^. Briefly, 45 mmol (1.8 g) of NaOH dissolved in 6 mL of water was mixed with 15 mL of ethanol (EtOH) and 30 mL of oleic acid (OA) under stirring. To the resulting mixture were selectively added 0.95 mmol (288 mg) of Y(Cl)_3_·6H_2_O, 0.05 mmol (18 mg) of Eu(Cl)_3_·6H_2_O and 10.2 mmol (377 mg) of NH_4_F dissolved in 4 mL of water. The solution was then transferred into an autoclave and heated at 200 °C for 24 h under stirring. The resulting nanoparticles were washed with water and ethanol several times, vacuum-dried, and collected as a white powder. For the optical experiments, a surface functionalization by ligand exchange was performed to ensure the good dispersion in polar solvents^78^. In total, 20 mg of NaYF_4_:Eu particles with native OA ligands were mixed with 2 mL of aqueous citrate solution (0.2 M) and the mixture was sonicated and centrifuged. The same cycle was repeated for three times to fully exchange OA with citrate. The residual OA in solution was removed by washing with EtOH and water. The resultant citrate-functionalized nanorods were well dispersed in water or ethylene glycol. The aqueous colloidal solution was spin-coated on a glass or quartz coverslip for the optical measurement of in-plane oriented nanorods (Figs. 2i, 4a, c–e). For the optical measurement of randomly oriented nanorods (Figs. 3c–e, 4b), polyvinyl alcohol (MW ~ 22000) was dissolved (0.1 g/mL) in the aqueous nanorod solution and then drop-casted on a glass coverslip embedding nanorods in a thick polymer film.

### Alignment of nanorods under electric field

For the measurement of the PL spectra in the π, σ, and α configurations (Fig. 2h), nanorods were aligned using electro-optical (E/O) cells (Supplementary Fig. 4). For the in-plane alignment (π and σ configurations), a lab-made in-plane switching cell was made with gold thin film electrodes deposited with a narrow gap (width ~ 150 µm) at the center. A spacer (thickness ~ 60 µm) was made at the border of the cell. In total, 20 µL of nanorods dispersion in ethylene glycol was dropped at the center and covered with a glass coverslip. An AC electric field (1 kHz, 0.47 V/µm) was applied to fully align the nanorods between the gap. For the homeotropic alignment (α configuration), a commercial homeotropic E/O cell (INSTEC) was used also with an AC electric field applied (500 kHz, 0.25 V/µm).

### Characterizations

High-resolution transmission electron microscopy (HR-TEM) was performed using JEOL JEM-2010F. Scanning electron microscopy (SEM) was performed using Hitachi S4800. Photoluminescence (PL) spectra of NaYF_4_:Eu single nanorods (Figs. 3c–e,  4a, b, Supplementary Figs. 4, 7, 8, Supplementary Data 1) were obtained using a upright polarizing optical microscope (Olympus BX 51WI, Objective: magnification = 100x, NA = 0.9). A fiber-coupled monochromatic excitation laser (OXXIUS, *λ*_center_ = 394 nm, FWHM = 0.7 nm) was used as an excitation source matching with the ^7^F_0_ → ^5^L_6_ transition of Eu^3+^. PL signals were collected with a fiber-coupled spectrometer (SpectraPro-300i, Princeton Instrument equipped with LN/CCD-1100-PP camera) or with a CCD camera (Discovery plus DTA DX 1600E SN, Fig. 3c-inset). PL signals of aligned nanorods under electric field (Fig. 2h, Supplementary Fig. 1,4) were obtained also using same optical setup with lower magnification (objective: 20x, NA = 0.45). The optical setup is schematized in Fig. 3c. The series of PL spectra with continuous analyzer angles were collected using an inverted microscope (Nikon Eclipse Ti) and two objectives: A Nikon CFI S Plan Fluor (Magnification = 40x, NA = 0.6 for Figs. 2i, 4c–e, Supplementary Figs. 12–14) and a Nikon CFI APO TIRF (Magnification = 60x, NA = 1.49 for Supplementary Fig. 14). The optical setup is equipped with a polarizer-mounted rotation stage (Newport, PR50CC), a piezoelectric stage (Mad Labs City, Model Nano-LP100), a spectrometer (Andor Shamrock 303i, Andor Newton 920 CCD camera) and a silicon avalanche photodiode (APD, Perkin Elmer, SPCM-AQRH-15). The scanned nanorod image (Fig. 2i-inset) was obtained using the synchronized APD and piezoelectric stage.

### Data treatment

Baselines of all the photoluminescence spectra were taken as a flat line with averaged intensity between 600 nm and 608 nm where Eu^3+^ luminescence is not present (Supplementary Fig. 9). Reference spectra used for the orientation calculation is normalized, as shown in Supplementary Fig. 16. Spectral fitting analysis was conducted on the wavelength ranges of 587.33–601.65 nm (MD) and 607.05–623.87 nm (ED). Area under curve ratiometry analysis was conducted on the four wavelength ranges of 587.75–592.85 nm (MD1), 592.85–598.5 nm (MD2), 610.00–617.75 nm (ED1), and 617.75–622.50 nm (ED2). The polar diagrams (Fig. 4c, d) were corrected to compensate the polarization-dependent beam distortion by optical parts. Detailed correction method using unpolarized white light background source is shown in the S.I. (Supplementary Fig. 17).

## Supplementary information

Supplementary Information

Description of Additional Supplementary Files

Supplementary Data 1

## Data Availability

The authors declare that the main data supporting the proposed methods are available within the article and its Supplementary Information files. Extra data are available from the corresponding author upon reasonable request.

## References

[1] Huang B, Bates M, Zhuang X (2009). Super-resolution fluorescence microscopy. Annu. Rev. Biochem..

[2] Yildiz A (2003). Myosin V walks hand-over-hand: single fluorophore imaging with 1.5-nm localization. Science.

[3] Weiss S (2000). Measuring conformational dynamics of biomolecules by single molecule fluorescence spectroscopy. Nat. Struct. Biol..

[4] Zhang X, Xiao Y, Qian X (2008). A ratiometric fluorescent probe based on FRET for imaging Hg2+ ions in living cells. Angew. Chem. Int. Ed..

[5] Saha K, Agasti SS, Kim C, Li X, Rotello VM (2012). Gold nanoparticles in chemical and biological sensing. Chem. Rev..

[6] Mayer KM (2008). A label-free immunoassay based upon localized surface plasmon resonance of gold nanorods. ACS Nano..

[7] Anker JN (2008). Biosensing with plasmonic nanosensors. Nat. Mater..

[8] Sosa H, Peterman EJG, Moerner WE, Goldstein LSB (2001). ADP-induced rocking of the kinesin motor domain revealed by single-molecule fluorescence polarization microscopy. Nat. Struct. Biol..

[9] Adachi K (2000). Stepping rotation of F1-ATPase visualized through angle-resolved single-fluorophore imaging. Proc. Natl Acad. Sci..

[10] Ha T, Laurence TA, Chemla DS, Weiss S (1999). Polarization spectroscopy of single fluorescent molecules. J. Phys. Chem. B.

[11] Royer CA (2006). Probing protein folding and conformational transitions with fluorescence. Chem. Rev..

[12] O’Neill M, Kelly SM (2011). Ordered materials for organic electronics and photonics. Adv. Mater..

[13] Mohraz A, Solomon MJ (2005). Direct visualization of colloidal rod assembly by confocal microscopy. Langmuir.

[14] Rosenberg SA, Quinlan ME, Forkey JN, Goldman YE (2005). Rotational motions of macro- molecules by single-molecule fluorescence microscopy. Acc. Chem. Res..

[15] Shroder DY, Lippert LG, Goldman YE (2016). Single molecule optical measurements of orientation and rotations of biological macromolecules. Methods Appl. Fluorescence.

[16] Backer AS, Lee MY, Moerner WE (2016). Enhanced DNA imaging using super-resolution microscopy and simultaneous single-molecule orientation measurements. Optica.

[17] Valades Cruz CA (2016). Quantitative nanoscale imaging of orientational order in biological filaments by polarized superresolution microscopy. Proc. Natl Acad. Sci..

[18] Lethiec C (2014). Measurement of three-dimensional dipole orientation of a single fluorescent nanoemitter by emission polarization analysis. Phys. Rev. X.

[19] Ohmachi M (2012). Fluorescence microscopy for simultaneous observation of 3D orientation and movement and its application to quantum rod-tagged myosin V. Proc. Natl Acad. Sci..

[20] Early KT (2009). Linear dipole behavior in single CdSe-Oligo(phenylene vinylene) nanostructures. ACS Nano..

[21] Chung I, Shimizu KT, Bawendi MG (2003). Room temperature measurements of the 3D orientation of single CdSe quantum dots using polarization microscopy. Proc. Natl Acad. Sci..

[22] Empedocles SA, Neuhauser R, Bawendi MG (1999). Three-dimensional orientation measurements of symmetric single chromophores using polarization microscopy. Nature.

[23] Brasselet S (2004). In situ diagnostics of the crystalline nature of single organic nanocrystals by nonlinear microscopy. Phys. Rev. Lett..

[24] Mayer L (2013). Single KTP nanocrystals as second-harmonic generation biolabels in cortical neurons. Nanoscale.

[25] Winters DG, Smith DR, Schlup P, Bartels RA (2012). Measurement of orientation and susceptibility ratios using a polarization-resolved second-harmonic generation holographic microscope. Biomed. Opt. Express.

[26] Tiaho F, Recher G, Rouède D (2007). Estimation of helical angles of myosin and collagen by second harmonic generation imaging microscopy. Opt. Express.

[27] Stoller P, Reiser KM, Celliers PM, Rubenchik AM (2002). Polarization-modulated second harmonic generation in collagen. Biophysical J..

[28] Rodríguez-Sevilla P (2016). Determining the 3D orientation of optically trapped upconverting nanorods by in situ single-particle polarized spectroscopy. Nanoscale.

[29] Green KK, Wirth J, Lim SF (2017). Nanoplasmonic upconverting nanoparticles as orientation sensors for single particle microscopy. Sci. Rep..

[30] Forkey JN, Quinlan ME, Alexander Shaw M, Corrie JET, Goldman YE (2003). Three-dimensional structural dynamics of myosin V by single-molecule fluorescence polarization. Nature.

[31] Yamamoto J (2015). Rotational diffusion measurements using polarization-dependent fluorescence correlation spectroscopy based on superconducting nanowire single-photon detector. Opt. Express.

[32] Tsay JM, Doose S, Weiss S (2006). Rotational and translational diffusion of peptide-coated CdSe/CdS/ZnS nanorods studied by fluorescence correlation spectroscopy. J. Am. Chem. Soc..

[33] Sezgin E (2019). Measuring nanoscale diffusion dynamics in cellular membranes with super-resolution STED–FCS. Nat. Protoc..

[34] Eggeling C, Widengren J, Rigler R, Seidel CAM (1998). Photobleaching of fluorescent dyes under conditions used for single-molecule detection: evidence of two-step photolysis. Anal. Chem..

[35] Song L, Hennink EJ, Young IT, Tanke HJ (1995). Photobleaching kinetics of fluorescein in quantitative fluorescence microscopy. Biophysical J..

[36] Shimizu KT (2001). Blinking statistics in single semiconductor nanocrystal quantum dots. Phys. Rev. B.

[37] Galland C (2011). Two types of luminescence blinking revealed by spectroelectrochemistry of single quantum dots. Nature.

[38] Hoogenboom JP, Hernando J, van Dijk EMHP, van Hulst NF, García-Parajó MF (2007). Power-law blinking in the fluorescence of single organic molecules. ChemPhysChem.

[39] Chen H-Y, Yang Y-C, Lin H-W, Chang S-C, Gwo S (2008). Polarized photoluminescence from single GaN nanorods: effects of optical confinement. Opt. Express.

[40] Lieb MA, Zavislan JM, Novotny L (2004). Single-molecule orientations determined by direct emission pattern imaging. J. Opt. Soc. Am. B.

[41] Patra D, Gregor I, Enderlein J (2004). Image analysis of defocused single-molecule images for three-dimensional molecule orientation studies. J. Phys. Chem. A.

[42] Li T (2012). Three-dimensional orientation sensors by defocused imaging of gold nanorods through an ordinary wide-field microscope. ACS Nano..

[43] Lethiec C (2014). Polarimetry-based analysis of dipolar transitions of single colloidal CdSe/CdS dot-in-rods. N. J. Phys..

[44] Böhmer M, Enderlein J (2003). Orientation imaging of single molecules by wide-field epifluorescence microscopy. J. Opt. Soc. Am. B.

[45] Lukosz W (1981). Light emission by multipole sources in thin layers. I. radiation patterns of electric and magnetic dipoles. J. Opt. Soc. Am..

[46] Kim J (2017). Monitoring the orientation of rare-earth-doped nanorods for flow shear tomography. Nat. Nanotechnol..

[47] Binnemans K (2015). Interpretation of europium(III) spectra. Coord. Chem. Rev..

[48] Brecher C, Samelson H, Lempicki A, Riley R, Peters T (1967). Polarized spectra and crystal-field parameters of Eu+3 in YVO4. Phys. Rev..

[49] Yang D (2019). Anisotropic excitation polarization response from a single white light-emitting β-NaYF4:Yb3+, Pr3+ microcrystal. Small.

[50] Li Z, Zhang Y, Jiang S (2008). Multicolor core/shell-structured upconversion fluorescent nanoparticles. Adv. Mater..

[51] Zhou J (2013). Ultrasensitive polarized up-conversion of Tm3+–Yb3+ doped β-NaYF4 single nanorod. Nano Lett..

[52] Yi G (2004). Synthesis, characterization, and biological application of size-controlled nanocrystalline NaYF4:Yb,Er infrared-to-visible up-conversion phosphors. Nano Lett..

[53] Zhou S (2013). Upconversion luminescence of NaYF4: Yb3+, Er3+ for temperature sensing. Opt. Commun..

[54] Vetrone F (2010). Temperature sensing using fluorescent nanothermometers. ACS Nano.

[55] Wang F (2010). Simultaneous phase and size control of upconversion nanocrystals through lanthanide doping. Nature.

[56] Thiriet, M. Nanobâtonnets de NaYF4 à upconversion: synthèse, dispersion colloïdale et propriétés électro-optiques, PhD thesis, Université Paris-Saclay, (2016).

[57] Görller-Walrand, C. & Binnemans, K. In *Handbook on the physics and chemistry of rare earths* Vol. 23, 121–283 (Elsevier, 1996).

[58] Powell, R. C. *Symmetry, group theory, and the physical properties of crystals*. Vol. 824 (Springer-Verlag New York, 2010).

[59] Atkins, P. W., Child, M. S. & Phillips, C. S. G. *Tables for group theory*. Vol. 6 (Oxford University Press Oxford, 1970).

[60] Tu D (2013). Breakdown of crystallographic site symmetry in lanthanide-doped NaYF4 crystals. Angew. Chem. Int. Ed..

[61] Gao D (2011). Efficient fluorescence emission and photon conversion of LaOF:Eu3+ nanocrystals. Appl. Phys. Lett..

[62] Sayre EV, Freed S (1956). Spectra and quantum states of the europic ion in crystals. II. fluorescence and absorption spectra of single crystals of europic ethylsulfate nonahydrate. J. Chem. Phys..

[63] Görller‐Walrand C (1992). Optical spectra and crystal‐field analysis of europium double nitrates. J. Chem. Phys..

[64] Chaudan E (2018). Polarized Luminescence of anisotropic LaPO4:Eu nanocrystal polymorphs. J. Am. Chem. Soc..

[65] Chacon R (2020). Measuring the magnetic dipole transition of single nanorods by spectroscopy and fourier microscopy. Phys. Rev. Appl..

[66] Kim J (2014). Optimized combination of intrinsic and form birefringence in oriented LaPO4 nanorod assemblies. Appl. Phys. Lett..

[67] Kim J (2012). LaPO4 mineral liquid crystalline suspensions with outstanding colloidal stability for electro-optical applications. Adv. Funct. Mater..

[68] Hänninen, P. & Härmä, H. *Lanthanide luminescence: photophysical, analytical and biological aspects*. Vol. 7 (Springer Science & Business Media, 2011).

[69] Labrador-Páez L (2018). Reliability of rare-earth-doped infrared luminescent nanothermometers. Nanoscale.

[70] Shen Y, Lifante J, Fernández N, Jaque D, Ximendes E (2020). In vivo spectral distortions of infrared luminescent nanothermometers compromise their reliability. ACS Nano.

[71] Rodríguez-Sevilla P (2016). Optical torques on upconverting particles for intracellular microrheometry. Nano Lett..

[72] Pierrat S, Hartinger E, Faiss S, Janshoff A, Sönnichsen C (2009). Rotational dynamics of laterally frozen nanoparticles specifically attached to biomembranes. J. Phys. Chem. C..

[73] Mor FM, Sienkiewicz A, Forró L, Jeney S (2014). Upconversion particle as a local luminescent brownian probe: a photonic force microscopy study. ACS Photonics.

[74] Chakrabarty A (2018). Nanofiber-directed anisotropic self-assembly of CdSe–CdS quantum rods for linearly polarized light emission evidenced by quantum rod orientation microscopy. Small.

[75] Smalyukh II, Shiyanovskii SV, Lavrentovich OD (2001). Three-dimensional imaging of orientational order by fluorescence confocal polarizing microscopy. Chem. Phys. Lett..

[76] Wang L, Li Y (2006). Na(Y1.5Na0.5)F6 single-crystal nanorods as multicolor luminescent materials. Nano Lett..

[77] Wang X, Zhuang J, Peng Q, Li Y (2005). A general strategy for nanocrystal synthesis. Nature.

[78] Cao T (2010). Water-soluble NaYF4:Yb/Er upconversion nanophosphors: synthesis, characteristics and application in bioimaging. Inorg. Chem. Commun..

