# Supplementary material for: Measuring 3D orientation of nanocrystals via polarized luminescence of rare-earth dopants
Source: Nat Commun. 2021 Mar 29;12:1943. doi: 10.1038/s41467-021-22158-4 (PMC8007814; doi:10.1038/s41467-021-22158-4)
Supplement: Supplementary file 1 — Supplementary Information [file 41467_2021_22158_MOESM1_ESM.pdf]

Supplementary Information

Measuring 3D Orientation of Nanocrystals via Polarized Luminescence of Rare-Earth Dopants

Jeongmo Kim[1], Reinaldo Chacón[2], Zijun Wang[1], Eric Larquet[1], Khalid Lahlil[1], Aymeric Leray[2], Gérard Colas-des-Francs[2], Jongwook Kim[1], and Thierry Gacoin[1]*

[1]Laboratoire de Physique de la Matière Condensée, CNRS, École Polytechnique, Institut Polytechnique de Paris, 91128 Palaiseau, France

[2]Laboratoire Interdisciplinaire Carnot de Bourgogne (ICB), UMR 6303 CNRS, Université Bourgogne Franche-Comté, 9 Avenue Savary, BP 47870, 21078 Dijon cedex, France

*E-mail: jong-wook.kim@polytechnique.edu

Supplementary Information includes

- Comparison of proposed method with pre-existing orientation analysis methods (page 3) → Supplementary Table 1
- Unpolarized feature of the α-configuration of $NaYF_4$:Eu nanorod (page 4) → Supplementary Figure 1
- Size and XRD analysis of $NaYF_4$:Eu nanorod (page 4) → Supplementary Figure 2-3
- Electro-optical switching experiment (page 5) → Supplementary Figure 4
- Full emission spectrum of $NaYF_4$:Eu nanorod (page 6) → Supplementary Figure 5
- Graphic explanation of Equation 2 and Equation 3 (page 6) → Supplementary Figure 6
- Orientation analysis using either electric dipole (ED) or magnetic dipole (MD) emission (page 7) → Supplementary Figure 7
- Analyzed 3D orientation of several $NaYF_4$:Eu nanorods (page 8) → Supplementary Figure 8
- Coordinates conversion equations (page 9) → Supplementary Equation 1-5
- Errors in orientation analysis (page 10-13) → Supplementary Figure 9-11
- Polar diagrams with highly varying global intensity (page 13) → Supplementary Figure 12
- DOP analysis of polarized photoluminescence of $NaYF_4$:Eu particles in different sizes and using different numerical aperture of objective (page 14-16) → Supplementary Figure 13-14, Supplementary Table 2-3
- Method of mirror angle discrimination (page 17-18) → Supplementary Figure 15
- Applicability of other lanthanide ions to the proposed method (page 19) → Supplementary Table 4
- Reference spectra normalization (page 20) → Supplementary Figure 16
- Polarization-dependency correction using white light source (page 21) → Supplementary Figure 17

Comparison of proposed method with pre-existing orientation analysis methods

Supplementary Table 1. Comparison of pre-existing orientation analysis method with the proposed method.

Technique	Fluorescence polarization microscopy using multiple dipoles (this work)	Fluorescence polarization microscopy using a single dipole	Back-focal plane imaging / defocusing imaging	Non-linear microscopy	Plasmonic imaging
Probe Material	Rare-earth-doped nanocrystal	Molecular phosphor / Quantum rod	Molecular phosphor / Quantum rod	Molecular or nanocrystal host of non-linear luminescence: two-photon fluorescence (TPF), second harmonic generation (SHG), etc.	Plasmonic nanoparticle
Analysis	Simultaneous consideration of polarization behaviors of multiple emission dipoles	Polarization behavior of a single emission dipole	Intensity distribution of emission dipole + Image analysis	Polarization behaviors of multiple non-linear processes	Polarization behavior of plasmonic light interaction
Pros	- Possible to measure 3D orientation angles with a single acquisition<br><br>- One measurement plane<br><br>- Robust to probe polydispersity and global intensity fluctuation (ratiometric analysis)<br><br>- No photobleaching or blinking	- Fast dynamics with molecular phosphors<br><br>- Wide range of phosphors can be used	- Possible to measure 3D orientation angles with a single acquisition<br><br>- One measurement plane<br><br>- Fast dynamics with molecular phosphors<br><br>- Wide range of phosphors can be used	- Possible to measure 3D orientation angles with a single acquisition<br><br>- One measurement plane.<br><br>- Probe-free measurement possible	- One measurement plane<br><br>- Fast optical process<br><br>- No photobleaching or blinking
Cons	- Relatively low temporal resolution due to low excitation cross-section and long decay time of lanthanide emission	- Complicated optical setup for multiple excitation angles and measurement planes<br><br>- Requires full scanning of polarization angles to measure 3D orientation angles<br><br>- Sensitive to photobleaching, blinking, or scattering<br><br>- Sensitive to experimental error and noise<br><br>- Sensitive to probe polydispersity	- Requires model-dependent image analysis<br><br>- Sensitive to photobleaching, blinking, or scattering<br><br>- Very sensitive to focusing	- Sensitive to external environment (optical index, temperature, etc)<br><br>- Complicated optical setup for non-linear microscopy<br><br>- Very sensitive to excitation condition	- Requires model-dependent image analysis for 3D orientation<br><br>- Very sensitive to polydispersity<br><br>- Sensitive to other sources of absorption and scattering
Literature (see the list at the end of the file)	1, 2, 3	4, 5, 6, 7, 8, 9, 10, 11, 12, 13, 14, 15, 16, 17, 18	19, 20, 21, 22, 23, 24, 25	26,27, 28, 29, 30	31, 32, 33, 34, 35, 36, 37, 38, 39

Unpolarized feature of the α-configuration of $NaYF_4$:Eu nanorod

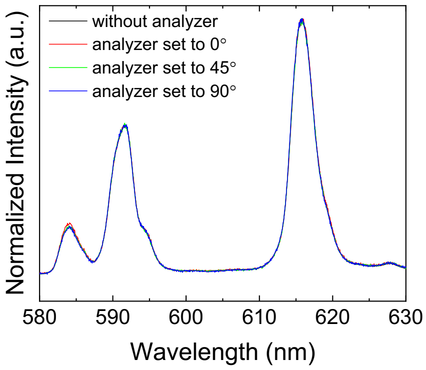

Supplementary Figure 1. Emission spectrum of α configuration measured without analyzer and with rotating analyzer (0°, 45°, 90°).

Size and XRD analysis of $NaYF_4$:Eu nanorod

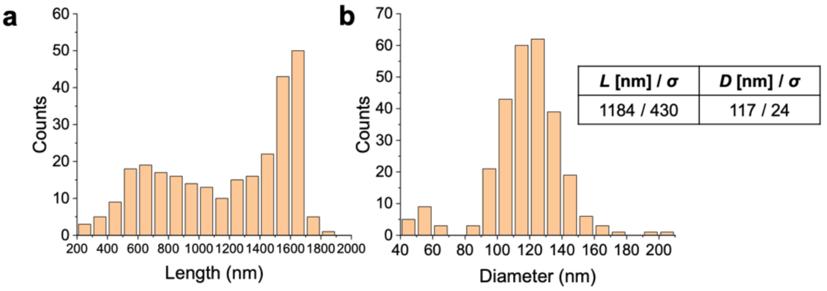

Supplementary Figure 2. Measured **(a)** length and **(b)** diameter distributions of $NaYF_4$:Eu nanorods. 276 rods were counted from SEM images. Inset table shows averaged length (L) and diameter (D) of $NaYF_4$:Eu nanorods with their standard deviations (σ).

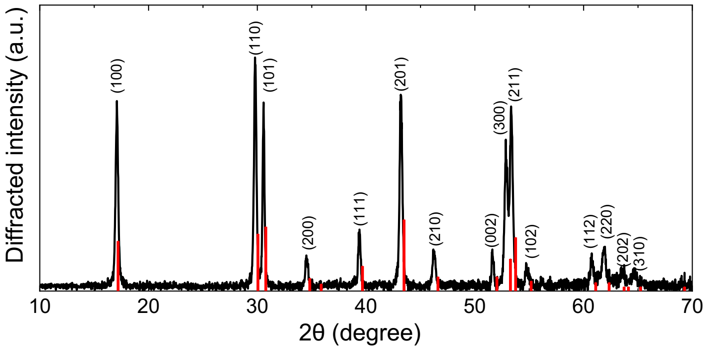

Supplementary Figure 3. XRD pattern of $NaYF_4$:Eu nanorods. Reference pattern of hexagonal β-$NaYF_4$ (JCPDS 16-0334) is displayed in red line.

Electro-optical switching experiment

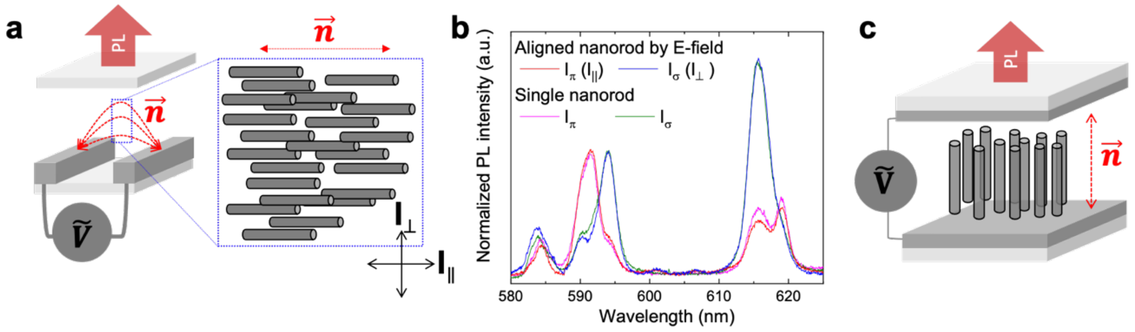

Supplementary Figure 4. **(a)** Schematic illustration of in-plane switching (IPS) electro-optical (E/O) cell. Red line stands for the direction of electric field (E-field) $\vec{n}$. Photoluminescence signal of π and σ reference configurations can be collected by putting analyzer parallel ($I_\parallel$) or perpendicular ($I_\perp$) to the direction of electric field $\vec{n}$, respectively. **(b)** Photoluminescence signal of π and σ reference configurations collected from aligned $NaYF_4$:Eu nanorod under electric field (E-field) and a single $NaYF_4$:Eu nanorod. **(c)** Schematic illustration of homeotropic orientation of nanorods modulated in the homeotropic E/O cell.

The reference spectra (π, σ and α) can be acquired using a single crystal or nematic LC state or uniformly aligned nanorods assembly under external field[1]. Here, we have applied alternating current (AC) electric field to align nanorods using electro-optical (E/O) cell with two geometries: in-plane switching (**Supplementary Figure 4a**) and homeotropic (**Supplementary Figure 4c**), which allow us to selectively measure the reference signals. Emission spectra of π and σ configuration presented in **Figure 2h** is collected using in-plane switching (IPS) E/O cell. The direction of electric field (E-field) $\vec{n}$ in IPS E/O cell is transverse at the middle of two electrodes (**Supplementary Figure 4a**). Emission spectrum of π and σ configurations can thus be collected by putting analyzer parallel ($I_\parallel$) or perpendicular ($I_\perp$) to the direction of E-field $\vec{n}$, respectively. As increasing the E-field intensity, the degree of alignment (order parameter) increases and shows eventual perfect alignment (1kHz, ~0.47 V/μm). Reference signals collected from aligned nanorods assembly is identical to that of single crystal (**Supplementary Figure 4b**), which confirms order parameter is close to 1 (perfect alignment).

The emission spectrum of α configuration (isotropic propagation along to the rod c-axis) of $NaYF_4$:Eu nanorods is obtained using a homeotropic electro-optical (E/O) cell (**Supplementary Figure 4c**). As increasing the E-field intensity, nanorods orients normal to the substrate. At eventual perfect alignment (500 kHz, ~0.25 V/μm), spectrum line shape is identical to that of π spectrum in $^5D_0$-$^7F_1$ MD transition and to that of σ spectrum in $^5D_0$-$^7F_2$ ED transition, which confirms the alignment is close to unity (**Figure 2h**).

Full emission spectrum of $NaYF_4$:Eu nanorod

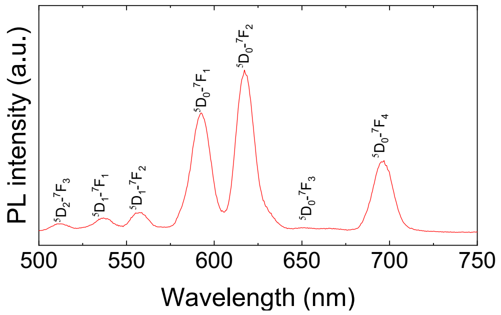

Supplementary Figure 5. Emission spectrum of β-$NaYF_4$:Eu crystals under the excitation at λ = ~394 nm.

Graphic explanation of Equation 2 and Equation 3

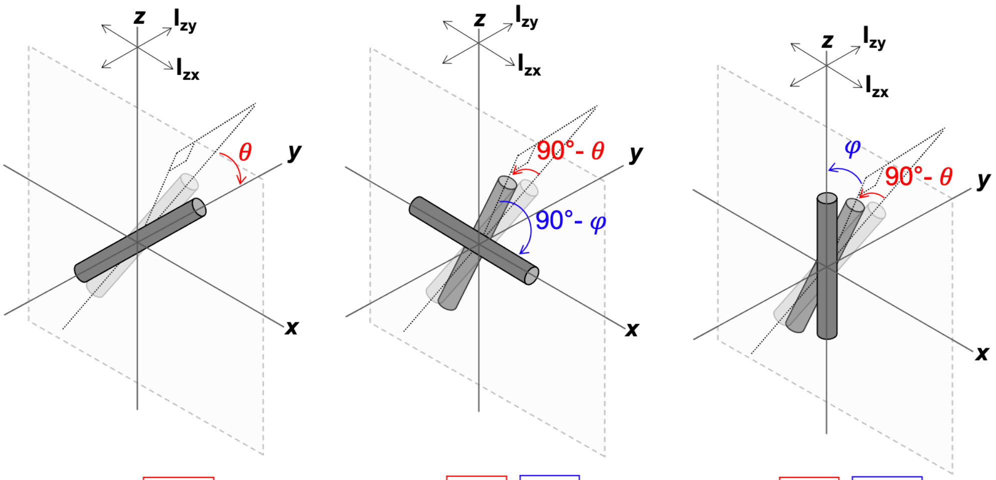

$$I_{zx} = I_\sigma \cdot \cos^2\theta + I_\pi \cdot \sin^2\theta \cdot \sin^2\varphi + I_\alpha \cdot \sin^2\theta \cdot \cos^2\varphi$$

$$I_{zy} = I_\pi \cdot \cos^2\theta + I_\sigma \cdot \sin^2\theta \cdot \sin^2\varphi + I_\alpha \cdot \sin^2\theta \cdot \cos^2\varphi$$

Supplementary Figure 6. Graphic explanation of the origin of **Equation 2** and **Equation 3** in the main text. Emission intensities collected at two orthogonal polarization angles (I_{zx} and I_{zy}) are represented by projected sum of the π, σ and α configurations.

Orientation analysis using either electric dipole (ED) or magnetic dipole (MD) emission

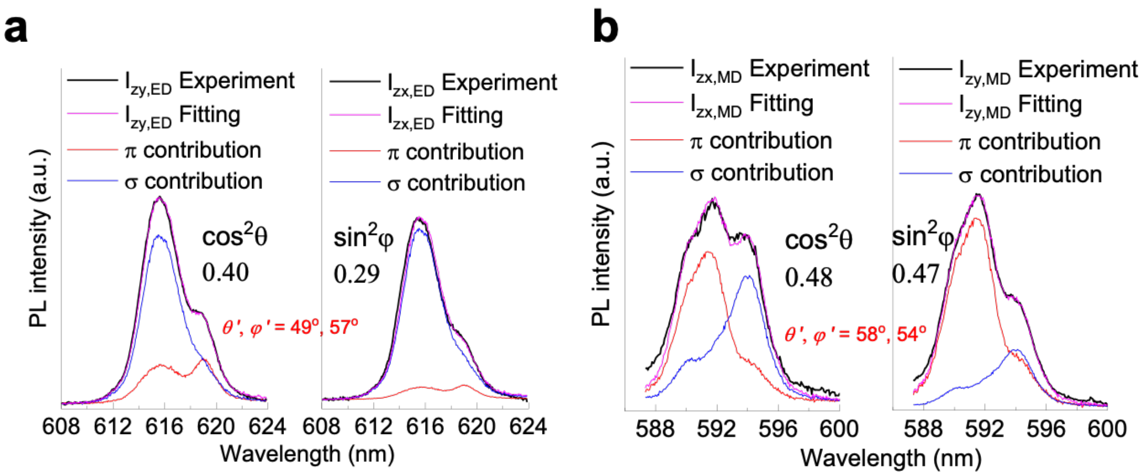

Supplementary Figure 7. Spectral fitting orientation analysis on single dipole emission from **(a)** ED and **(b)** MD using two orthogonal polarizations (I_{zx}, I_{zy}) of a nanorod presented in **Figure 3c-e**. The calculated values of the trigonometric functions of (θ, φ) and the absolute values of (θ', φ') are presented in the figure.

Analyzed 3D orientation of several $NaYF_4$:Eu nanorods

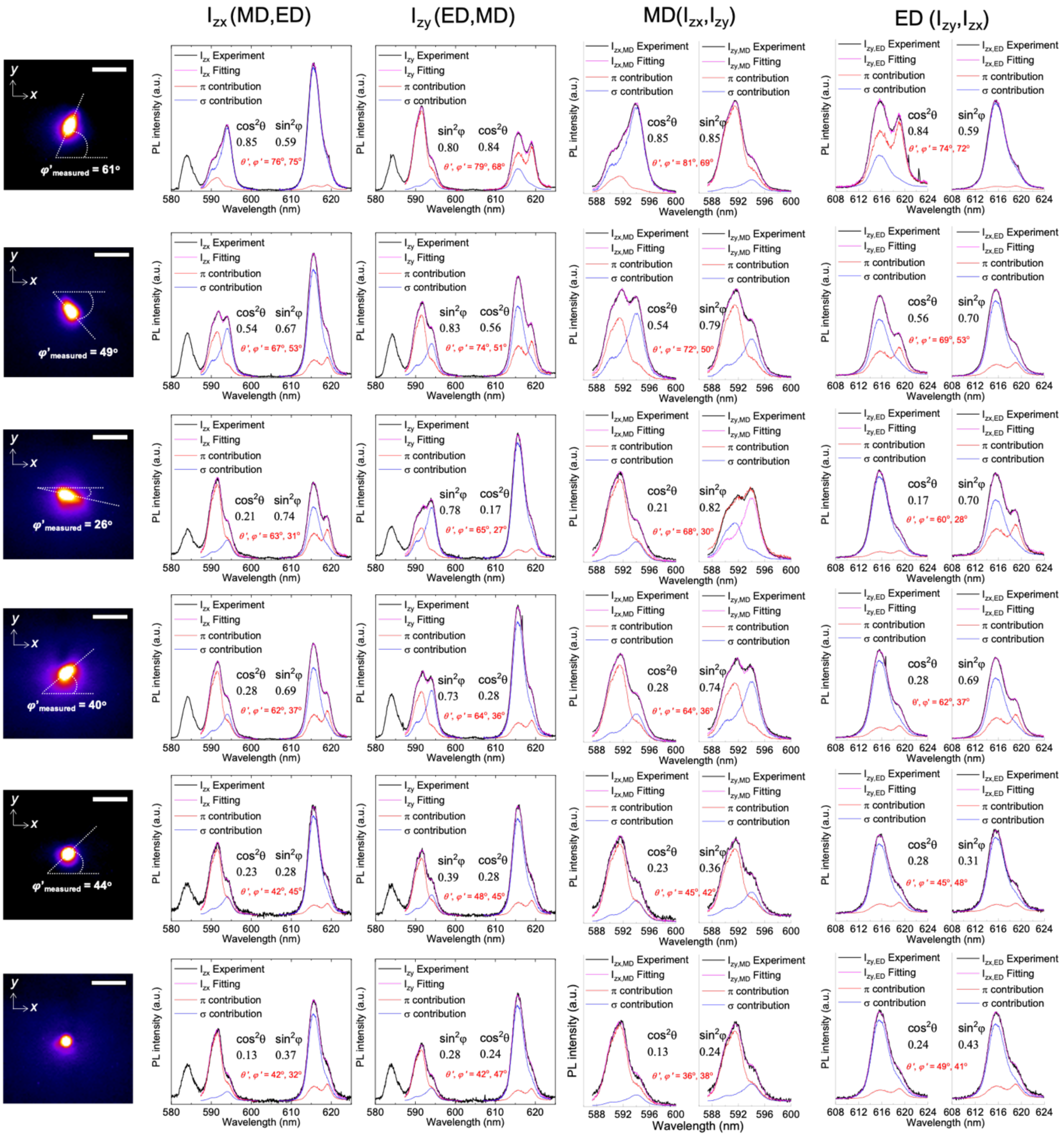

Supplementary Figure 8. Spectral fitting orientation analysis of 6 different $NaYF_4$:Eu nanorods randomly oriented in polymer film. First column displays a photograph of the photoluminescence (PL) of a single nanorod captured by the CCD camera (scale bar: 2 μm). Spectral fitting analysis using a polarized PL spectrum of I_{zx} (second column), I_{zy} (third column) where both MD and ED emissions are used for the orientation analysis. Spectral fitting analysis using a single dipole emission of MD (fourth column) and ED (fifth column) where two orthogonal polarizations (I_{zx}, I_{zy}) are used for the orientation analysis. The calculated values of the trigonometric functions of (θ, φ) and the absolute values of (θ', φ') are presented in the figure.

Conversion from (θ, φ) to (θ', φ')

Spherical coordinates (1, θ, φ) of a nanorod can be expressed in cartesian coordinates system (x, y, z) (**Figure 3a, b**) as:

$x\ coordinate = \sin\theta \cdot \sin\varphi = \sin\theta' \cdot \cos\varphi'$ **Supplementary Equation (1)**

$y\ coordinate = \cos\theta = \sin\theta' \cdot \sin\varphi'$ **Supplementary Equation (2)**

$z\ coordinate = \sin\theta \cdot \cos\varphi = \cos\theta'$ **Supplementary Equation (3)**

Now, out-of-plane angle θ' can be calculated from determined angle set (θ, φ) using **Supplementary Equations 3** and the in-plane angle φ' can be calculated by using **Supplementary Equation 2,3**, as summarized in **Supplementary Equation 4,5**.

$\theta' = \cos^{-1}(\sin\theta \cdot \cos\varphi)$ **Supplementary Equation (4)**

$$\varphi' = \sin^{-1}\left(\cos\theta / \sin\theta'\right) = \sin^{-1}\left(\cos\theta / \sqrt{1 - \cos^2\theta'}\right) = \sin^{-1}\left(\cos\theta / \sqrt{1 - \sin^2\theta \cdot \cos^2\varphi}\right)$$

Supplementary Equation (5)

Errors in orientation analysis

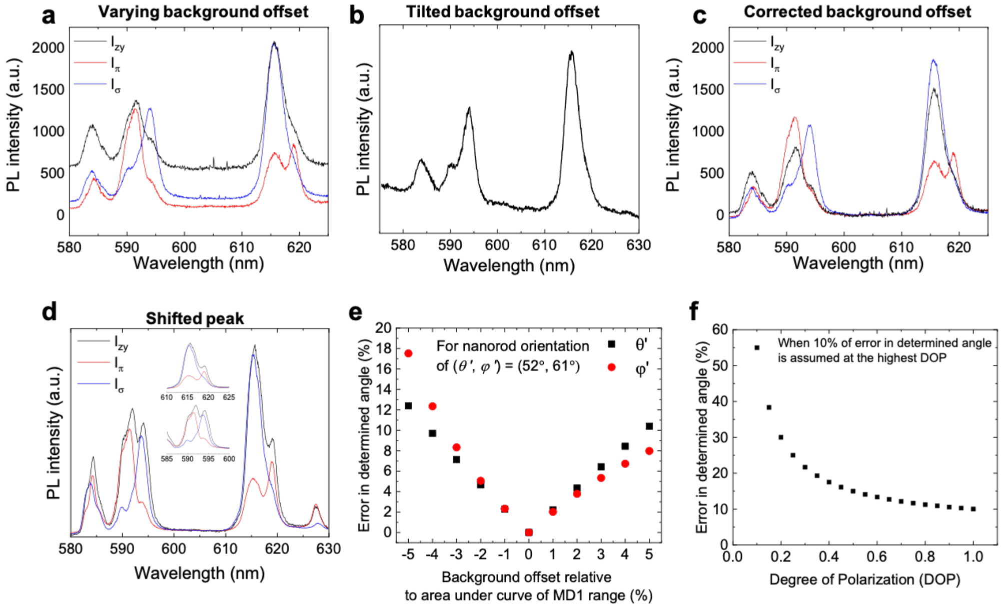

Supplementary Figure 9. Examples of experimental errors in background treatment: **(a)** varying background offset where the background offset of experimental and reference spectrum is not subtracted correctly, **(b)** titled background offset where the significant background signal from external source is added. **(c)** Corrected background for orientation analysis. **(d)** Example of experimental peak shift error where the wavelength of intensity peak of the experimental and reference spectrum do not match. **(e)** Simulated error in determined angle (θ', φ') as a function of amount of background offset relative to the area under curve of MD1 range. Simulation is made using the dataset of a certain orientation of a nanorod (θ', φ') = (52°, 61°). **(f)** Simulated error in determined angle as a function of degree of polarization (DOP). The Error in determined angle is assumed to have a 10 % when the DOP is highest (DOP = 1.0).

Supplementary Figure 9-11 discuss experimental errors that could deteriorate the accuracy of orientation analysis and its propagation to orientation determination. Orientation analysis can be degraded primarily for two reasons. The first reason is the background offset. **Supplementary Figure 9a** shows the varying background offset for the reference spectrum (red, blue line) and the experimental spectrum (black line). The difference in background offset can be attributed to the optical anisotropy of spectrometer gratings, different emission beam distortion as a function of analyzer angle, different focus of excitation to the sample, different spectral efficiency of the analyzer, *etc*. When the excitation/emission is not perfectly aligned/focused on the center of the emitters, emission beam may also be distorted due to the abrupt change in refractive index or wave-guiding effect, causing the background offset to be tilted as shown in **Supplementary Figure 9b**. The collection of autofluorescence from the substrate or any other surrounding components (due to UV-laser used for Eu^{3+} ions excitation) can be significant if the emission intensity of lanthanide ions is low. All of these sources affecting the background offset may degrade the precision of the orientation analysis. These backgrounds offset should be corrected by subtracting them, as shown in **Supplementary Figure 9c.** In this study, the baselines of the experimental spectra were taken as a flat line at the averaged intensity between 600 nm and 608 nm where Eu^{3+} luminescence is not present, which is an approximation that may have caused certain errors. Other wavelengths can be chosen as preferred in their experimental availability. It is also interesting to note that the effect of autofluorescence on the background offset can be technically minimized by using higher wavelength excitation

(e.g. 464 nm for Eu^{3+} ions excitation instead of 394 nm used in this study) or using a time-gated collection (decay of autofluorescence is on the scale of ~μs and the decay of lanthanide ions is on the scale of ~ms). The second possible reason is the peak shift. **Supplementary Figure 9d** represents an example of peak shift between the reference spectra (red, blue line) and an experimental spectrum (black line). Such small peak shifts can occur if the optical environment of the phosphor is changed or when the spectrometer grating was moved in the meantime. Although the shift is small, it may affect significantly on the ratiometric analysis. Therefore, the reference and experimental spectra should be collected under exactly the same condition to avoid this source of errors.

As noted above, if the background offset is not handled properly, it could degrade the accuracy of the orientation analysis. **Supplementary Figure 9e** shows the simulated error in (θ', φ') as a function of the amount of background offset relative to the area under curve of MD1 range. The simulation is done using the dataset of a certain orientation of a nanorod which shows the calculated angle of (θ', φ') = (52°, 61°) when the background offset is subtracted correctly. When the 5% of background offset relative to the area under curve of MD1 range is subtracted less or more than necessary, the determined angle shows a deviation of 10~12 % for θ' and 8~18 % for φ' comparing to values calculated with the appropriate background treatment. However, note that these values of angle deviation cannot be considered as general values because the amount of error varies also depending on the absolute values of (θ', φ'). The errors are relatively large when θ' or φ' is close to 0° or to 90° (i.e. when the rods are oriented parallel or perpendicular to the analyzer or to the substrate) (see the discussion in **Supplementary Figure 10,11**).

Finally, the effect of degree of polarization (DOP) on the determination of the orientation is simulated (**Supplementary Figure 9f**). The accuracy of the orientation analysis depends on the DOP of the peaks considered for the analysis. It can be seen that when 10 % error in angle is assumed to occur when the DOP is highest (DOP = 1, perfect polarization), the error in determining angle increases to 15% when DOP is 0.5 and to 30% when DOP is 0.2. This result shows a quantitative relation between the DOP and accuracy of angular measurement. Finding a host crystal structure giving a higher DOP of emission is therefore important to improve the accuracy of the proposed method[40].

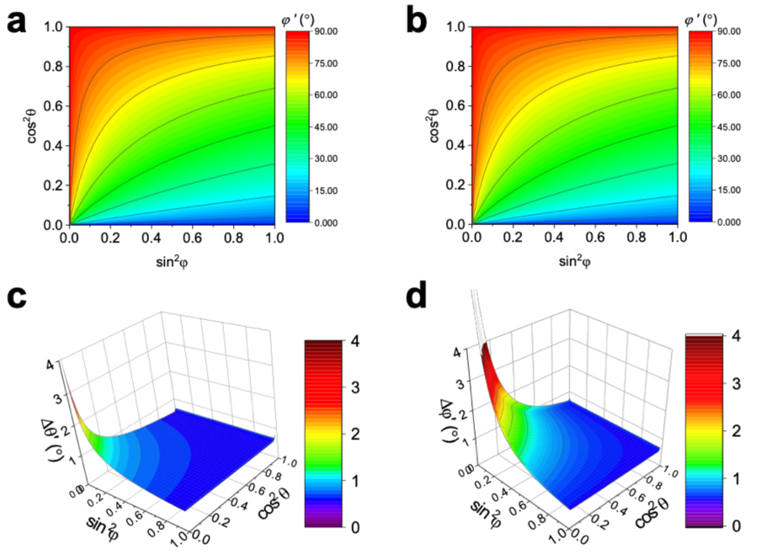

Supplementary Figure 10. Simulated **(a)** out-of-plane angle θ' and **(b)** in-plane angle φ' as a function of $\sin^2\varphi$ and $\cos^2\theta$ based on **Equations 8-9**. Simulated error Δ in **(c)** out-of-plane angle θ' and **(d)** in-plane angle φ' as a function of $\sin^2\varphi$ and $\cos^2\theta$. $\Delta\theta' = \sqrt{\Delta_{sin^2\varphi}\theta' \times \Delta_{cos^2\theta}\theta'}$ where $\Delta_{sin^2\varphi}\theta'$ and $\Delta_{cos^2\theta}\theta'$ are the deviated angle of θ' when $sin^2\varphi$ and $cos^2\theta$ deviates 0.02, respectively. $\Delta\varphi' = \sqrt{\Delta_{sin^2\varphi}\varphi' \times \Delta_{cos^2\theta}\varphi'}$ where $\Delta_{sin^2\varphi}\varphi'$ and $\Delta_{cos^2\theta}\varphi'$ are the deviated angle of φ' when $sin^2\varphi$ and $cos^2\theta$ deviates 0.02, respectively.

Supplementary Figure 10a-b shows the set of angles (θ', φ') calculated from the set of angles ($\cos^2\theta$, $\sin^2\varphi$) based on the **Equations 8-9**. Any experimental error in fitting analysis or area under curve ratiometry leads to the error in determining the set of angles ($\cos^2\theta$, $\sin^2\varphi$), which is the basis of conversion to (θ', φ'). Assuming that the experimental error in the determined set of angles ($\Delta\cos^2\theta$, $\Delta\sin^2\varphi$) is equal to 0.02, the error in the out-of-plane angle θ' ($\Delta\theta'$) and in-plane angle φ' ($\Delta\varphi'$) can be estimated, as shown in **Supplementary Figure 10c-d**. According to the simulated profile, it is more likely to have a larger error during the angle conversion when the rods are oriented close to normal to the measurement plane (θ'=0°). This can be explained by the fact that the line shape of polarized spectrum will not change much because the spectrum is dominated by α configuration which is an isotropic configuration (**Supplementary Figure 1**). The large error is also expected when rods are lying on the measurement plane (θ'= 90°) or oriented parallel or perpendicular to the analyzer angle (φ'= 0 ° or 90 °). This error is due to the shape of the square sinusoidal function which has a pole at 0 ° and 90 °. In particular, at this extreme condition, even for the very small error in determined angle (e.g. $\Delta\cos^2\theta = \Delta\sin^2\varphi = 10^{-3}$) the correct analysis could not be carried out because the calculated $\cos^2\theta$, $\sin^2\varphi$ can be off sinusoidal function range (e.g. $\cos^2\theta = \Delta\sin^2\varphi = 1+10^{-3}$).

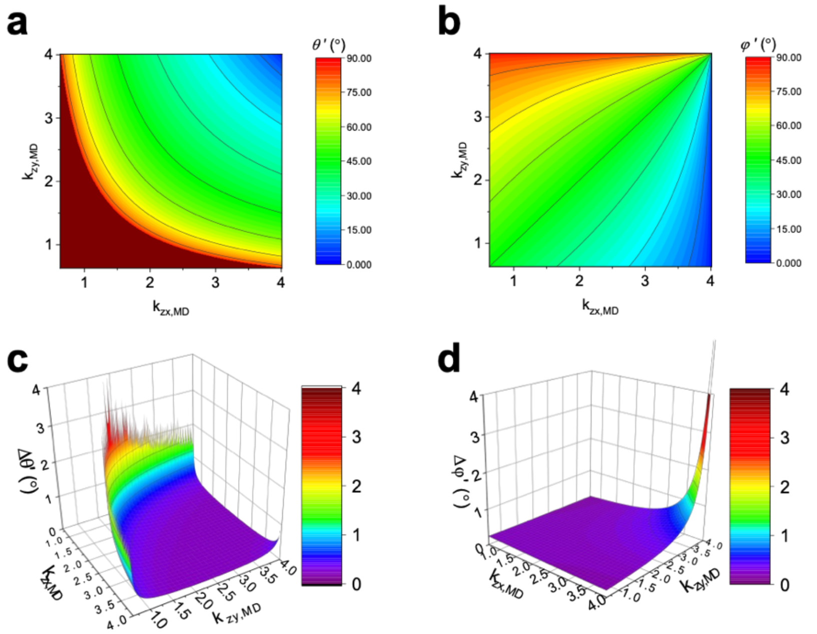

Supplementary Figure 11. Simulated **(a)** out-of-plane angle θ' and **(b)** in-plane angle φ' based on **Equation 8,9,12,13** as a function of area under curve ratio ($k_{zx,MD}$, $k_{zy,MD}$). Simulated error Δ in **(c)** out-of-plane angle θ' and **(d)** in-plane angle φ' as a function of ($k_{zx,MD}$, $k_{zy,MD}$). $\Delta\theta' = \sqrt{\Delta_{k_{zx,MD}}\theta' \times \Delta_{k_{zy,MD}}\theta'}$ where $\Delta_{k_{zx,MD}}\theta'$ and $\Delta_{k_{zy,MD}}\theta'$ are the deviated angle of θ' when $k_{zx,MD}$ and $k_{zy,MD}$ deviates 0.02, respectively. $\Delta\varphi' = \sqrt{\Delta_{k_{zx,MD}}\varphi' \times \Delta_{k_{zy,MD}}\varphi'}$ where $\Delta_{k_{zx,MD}}\varphi'$ and $\Delta_{k_{zy,MD}}\varphi'$ are the deviated angle of φ' when $k_{zx,MD}$ and $k_{zy,MD}$ deviates 0.02.)

The same error simulation illustrated in **Supplementary Figure 10** can be performed directly in the orientation analysis using an area under curve ratiometry approach. When one considers only the MD emission, one can calculate a set of angles (θ', φ') based on the area under curve ratio ($k_{zx,MD}$, $k_{zy,MD}$) of the polarized spectrum obtained at from two orthogonal polarizations according to **Equations 8-9** and **Equations 12-13 (Supplementary Figure 11a,b)**. Any error in obtaining ($k_{zx,MD}$, $k_{zy,MD}$) result in an error in determined angle. When the error in area under curve ratio ($\Delta k_{zx,MD}$, $\Delta k_{zy,MD}$) is assumed equal to 0.02, the error of calculated angle ($\Delta\theta'$, $\Delta\varphi'$) can be simulated, as shown in **Supplementary Figure 11c,d.** According to the simulated profile, the errors are relatively larger when θ' is close to 0° or to 90°, which is consistent with the discussion made in **Supplementary Figure 10**. As discussed above, even for the very small of error in determined angle due to the error in obtained area under curve ratio, the correct analysis could not be performed because the calculated $\cos^2\theta$, $\sin^2\varphi$ can be out of the sinusoidal function range.

Polar diagrams with highly varying global intensity

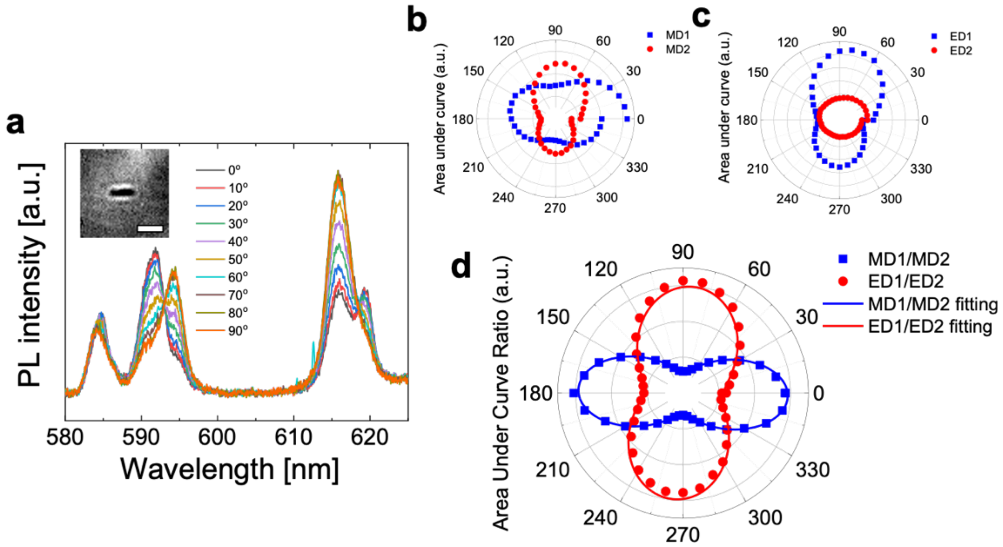

Supplementary Figure 12. **(a)** Polarized PL spectra of a single $NaYF_4$:Eu nanorod lying on a plane substrate with varied analyzer angles. The inset is an image of CCD captured nanorod by white-light illumination (scale bar = 3 μm). **(b-d)** Polar diagrams of the selected areas under curve for **(b)** MD1 and MD2, **(c)** ED1 and ED2, and **(d)** their ratios MD1/MD2 and ED1/ED2. The selected wavelength ranges (MD1, MD2, ED1, and ED2) to perform the area under curve ratiometry are highlighted in **Figure 2i**.

DOP analysis of polarized photoluminescence of $NaYF_4$:Eu particles in different sizes and using different numerical aperture of objective

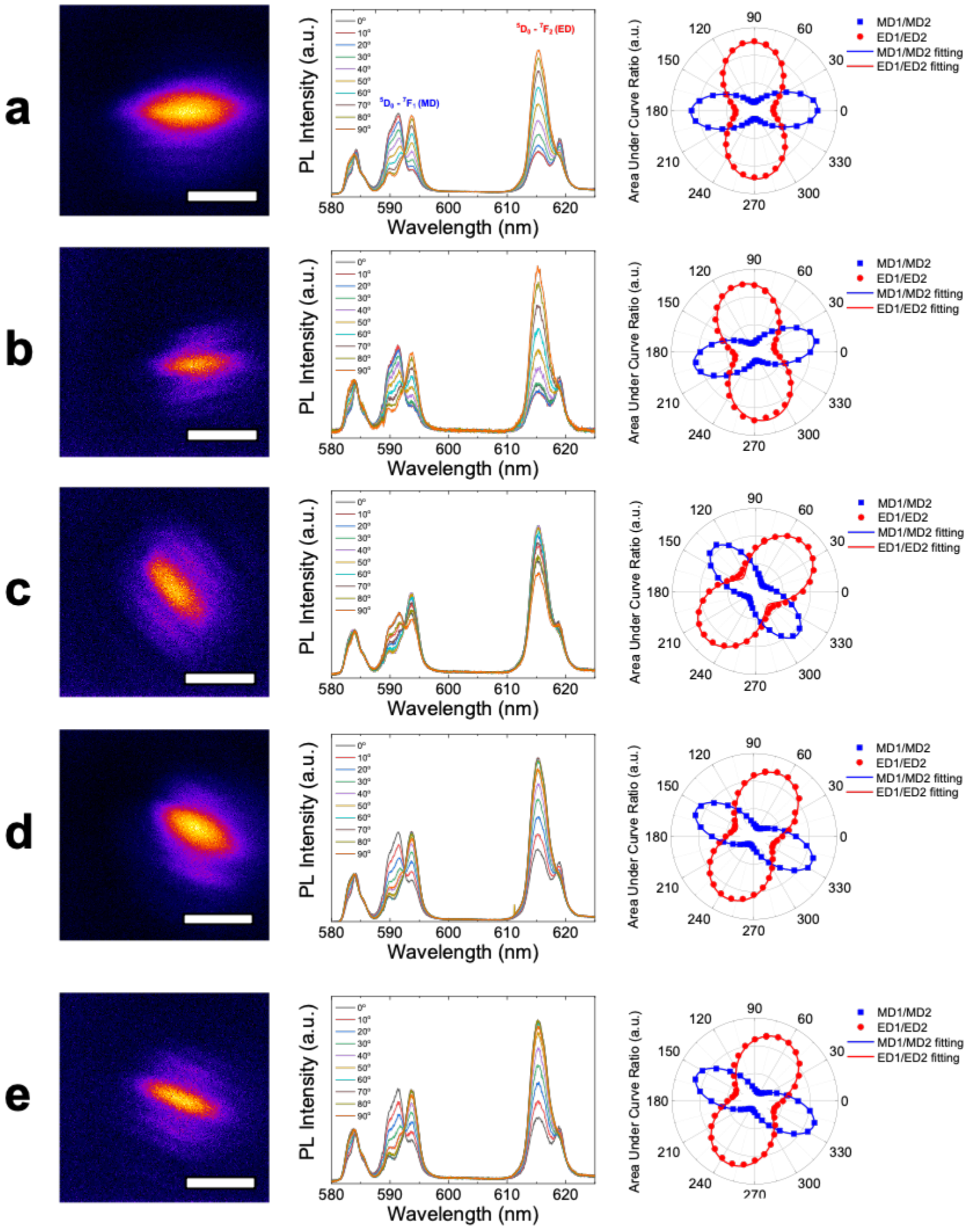

Supplementary Figure 13. **(a-e)** Polarized photoluminescence of five randomly selected $NaYF_4$:Eu nanorods with different sizes. First column shows an image of the nanorod obtained by scanning the PL signal using a piezo-stage (scale bar: 2 μm). Second column shows polarized PL spectra of corresponding $NaYF_4$:Eu nanorod lying on a plane substrate with varied analyzer angles. Third column shows polar diagrams of the selected areas under curve for the ratios of MD1/MD2 and ED1/ED2. The selected wavelength ranges (MD1, MD2, ED1, and ED2) to perform the area under curve ratiometry are highlighted in **Figure 2i**. Calculated DOPs of these ratios are presented in **Supplementary Table 2**.

Supplementary Table 2. Calculated DOPs of area under curve ratio (MD1/MD2 and ED1/ED2) of polarized PL spectra of $NaYF_4$:Eu nanorods with different sizes (**Supplementary Figure 13**). DOP is calculated according to **Equation 1**.

		I_π	I_σ	DOP		I_π	I_σ	DOP
Supplementary Figure 13a		4.57	0.60	0.77		1.34	4.94	-0.57
Supplementary Figure 13b		4.43	0.64	0.75		1.34	4.99	-0.58
Supplementary Figure 13c	MD1/MD2	4.35	0.66	0.74	ED1/ED2	1.34	4.92	-0.57
Supplementary Figure 13d		4.58	0.64	0.75		1.34	4.88	-0.57
Supplementary Figure 13e		4.59	0.63	0.76		1.34	4.91	-0.57

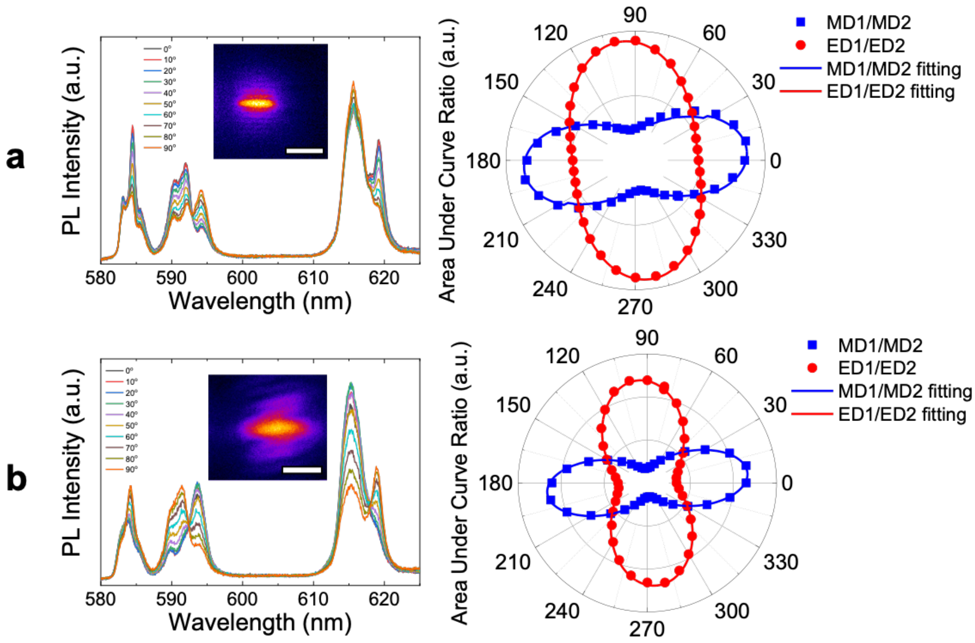

Supplementary Figure 14. **(a,b)** Polarized photoluminescence spectra of $NaYF_4$:Eu nanorod measured using objective numerical aperture of **(a)** 1.49 and **(b)** 0.6. First column shows the polarized PL spectra of a single $NaYF_4$:Eu nanorod lying on a plane substrate with varied analyzer angles. The inset is an image of the nanorod obtained by scanning the PL signal using a piezo-stage (scale bar: 2 μm). Second column shows a polar diagram of the selected areas under curve for the ratios of MD1/MD2 and ED1/ED2. The selected wavelength ranges (MD1, MD2, ED1, and ED2) to perform the area under curve ratiometry are highlighted in **Figure 2i**. Calculated DOPs of these ratios are presented in **Supplementary Table 3**.

Supplementary Table 3. Calculated DOPs of area under curve ratio (MD1/MD2 and ED1/ED2) of polarized PL spectra of $NaYF_4$:Eu nanorods measured using different numerical aperture (NA) of objective (**Supplementary Figure 14**). DOP is calculated according to **Equation 1**.

		I_π	I_σ	DOP		I_π	I_σ	DOP
NA 1.49 (Supplementary Figure 14a)	MD1/MD2	3.45	0.95	0.57	ED1/ED2	1.93	3.70	-0.31
NA 0.6 (Supplementary Figure 14ab)		4.67	0.66	0.75		1.34	4.79	-0.56

The detected line shape of polarized photoluminescence can vary depending on the numerical aperture (NA) of objective, as shown in **Supplementary Figure 14a,b**. Spectra are measured separately using a different particle that has a different size. As morphology doesn't affect the polarization behavior (**Supplementary Figure 13**, **Supplementary Table 2**), the difference of line shape and calculated DOP is purely due to the NA of the objective.

Method of mirror angle discrimination

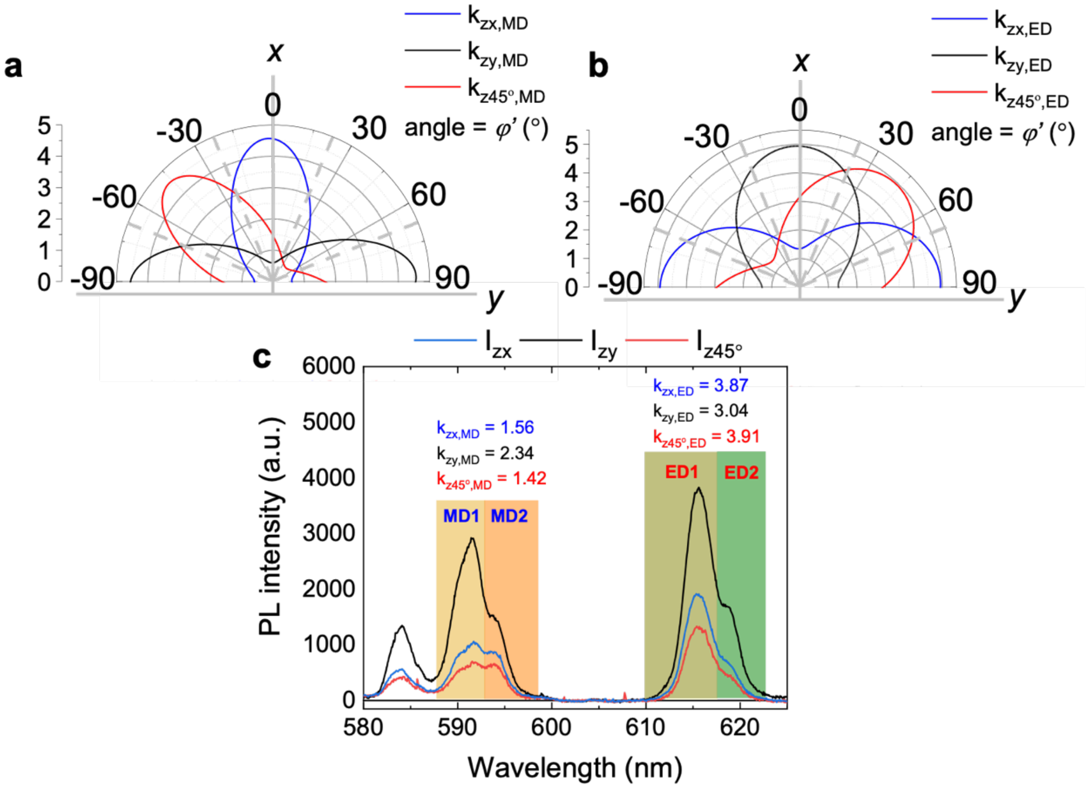

Supplementary Figure 15. The reference *k*-polar diagram plotted based on **Figure 4e** at different analyzer angle (k_{zx}, k_{zy} and $k_{z45°}$) within wavelength ranges of **(a)** MD and **(b)** ED as a function of in-plane angle φ'. **(c)** Polarized photoluminescence of a randomly oriented $NaYF_4$:Eu single nanorod measured at different analyzer angle (I_{zx}, I_{zy}, $I_{z45°}$). The selected wavelength ranges (MD1, MD2, ED1, and ED2) used for the area under curve ratiometry are highlighted in different colors. Measured area under curve ratio for MD and ED at different analyzer angle ($k_{zx,MD}$, $k_{zy,MD}$, $k_{z45°,MD}$, $k_{zx,ED}$, $k_{zy,ED}$, $k_{z45°,ED}$) are presented in the figure.

Once in-plane angle φ' is determined, mirror angle can be discriminated by a boundary condition established using reference *k*-polar diagram shown in **Supplementary Figure 15a, b**. Boundary condition can be written considering absolute value of *k* as :

for MD transition (**Supplementary Figure 15a**),

case 1: $0° < \varphi' < 22.5°$,

if $k_{z45°,MD} - k_{zy,MD} < 0.94$, then $\varphi' > 0°$

if $0.94 < k_{z45°,MD} - k_{zy,MD}$, then → $\varphi' < 0°$

case 2 : $22.5° < \varphi' < 67.5°$,

if $k_{z45°,MD} - k_{zx,MD} < 0$, then $\varphi' > 0°$

if $0 < k_{z45°,MD} - k_{zx,MD}$, then $\varphi' < 0°$

case 3: $67.5° < \varphi' < 90°$,

if $k_{z45°,MD} - k_{zx,MD} < 0.92$, then $\varphi' > 0°$

if $0.92 < k_{z45°,MD} - k_{zx,MD}$, then $\varphi' < 0°$

for ED transition (**Supplementary Figure 15b**),

case 1: $0° < \varphi' < 22.5°$,

if $k_{zy,ED} - k_{z45°,ED} < 1.8$, then $\varphi' > 0°$

if $1.8 < k_{z45°,ED} - k_{zy,ED}$, then $\varphi' < 0°$

case 2 : $22.5° < \varphi' < 67.5°$,

if $0 < k_{z45°,ED} - k_{zx,ED}$, then $\varphi' > 0°$

if $k_{z45°,ED} - k_{zx,ED} < 0$, then $\varphi' < 0°$

case 3: $67.5° < \varphi' < 90°$,

if $k_{z45°,ED} - k_{zx,ED} < 2.0$, then $\varphi' > 0°$

if $2.0 < k_{z45°,ED} - k_{zx,ED}$, then $\varphi' < 0°$

In the analyzed orientation shown in **Figure 3d,e**, in-plane angle φ' is determined to 59° from I_{zx} polarization and 52° from I_{zy} polarization, which are both the case 2. Measured *k* value at different analyzer angle ($k_{zx,MD}$, $k_{zy,MD}$, $k_{z45°,MD}$, $k_{zx,ED}$, $k_{zy,ED}$, $k_{z45°,ED}$) shown in **Supplementary Figure 15c** is thus to be served to decide whether the in-plane angle φ' is positive or negative, considering boundary condition written above. As $k_{z45°,MD} - k_{zx,MD}$ is negative and $k_{z45°,ED} - k_{zx,ED}$ is positive, in-plane angle φ' is determined to positive, which is consistent with CCD captured PL image (**Figure 3c**-inset). If the absolute value of in-plane angle φ' is close to boundary (e.g. 0°, 22.5°, 67.5° and 90°), small error in measured area under curve ratio *k* can result in wrong determination.

Applicability of other lanthanide ions to the proposed method

Supplementary Table 4. Summary of the transition of common lanthanide ions for spectroscopy (Tb^{3+}, Ho^{3+}, Er^{3+} and Tm^{3+}). The energy level of the transition, the wavelength of the transition and the nature of the transition are summarized. The nature of the transition is estimated according to the selection rule[41]. The magnetic dipole (MD) transition is blue, and the electric dipole (ED) transition is red.

Lanthanide ions	Transitions					
Tb^{3+}	$^5D_4$-$^7F_6$ (480 nm, ED)	$^5D_4$-$^7F_5$ (540 nm, MD)	$^5D_4$-$^7F_4$ (580 nm, MD)	$^5D_4$-$^7F_3$ (620 nm, MD)		
Ho^{3+}	$^5F_4$/$^5S_2$-$^5I_8$ (540 nm, ED)	$^5F_5$-$^5I_8$ (650 nm, ED)	$^5F_5$-$^5I_6$ (1487 nm, MD)	$^5I_6$-$^5I_8$ (1200 nm, ED)	$^5I_7$-$^5I_8$ (2000 nm, MD)	
Er^{3+}	$^2H_{11/2}$-$^4I_{15/2}$ (520 nm, ED)	$^4S_{3/2}$-$^4I_{15/2}$ (540 nm, ED)	$^4F_{9/2}$-$^4I_{15/2}$ (660 nm, ED)	$^4I_{11/2}$ -$^4I_{15/2}$ (980 nm, ED)	$^4I_{13/2}$ -$^4I_{15/2}$ (1520 nm, MD)	
Tm^{3+}	$^1I_6$-$^3H_6$ (290 nm, MD)	$^1I_6$-$^3F_4$ (340 nm, ED)	$^1D_2$-$^3H_6$ (360 nm, ED)	$^1D_2$-$^3F_4$ (450 nm, ED)	$^1G_4$-$^3H_6$ (475 nm, ED)	$^1G_4$-$^3F_4$ (640 nm, MD)
	$^3F_3$-$^3H_6$ (695 nm, ED)	$^1D_2$-$^3F_3$ (740 nm, MD)	$^1G_4$-$^3H_5$ (770 nm, MD)	$^3H_4$-$^3H_6$ (790 nm, ED)	$^1G_4$-$^3H_4$ (1170 nm, MD)	$^3H_4$-$^3F_4$ (1470 nm, MD)
			$^3F_4$-$^3H_6$ (1800 nm, ED)			

Supplementary Table 4 summarizes the nature and energy levels of the transitions of the lanthanide ions commonly used for spectroscopy (Tb^{3+}, Ho^{3+}, Er^{3+} and Tm^{3+}). The nature of the transition is estimated according to the selection rule[41] . We have shown that the 3D orientation of nanocrystals can be calculated by simultaneously taking into account the transition of MD and ED with a single viewing angle. According to **Supplementary Table 4**, the same analysis can be performed using other commonly used lanthanide ions because they also show a transition of both MD and ED. For example, when considering the use of Tb^{3+}, one can choose the transition of $^5D_4$-$^7F_6$ for ED emission and the transition of $^5D_4$-$^7F_5$ for MD emission for the orientation analysis. As these two transitions appear at adjacent wavelength ranges (480 nm for the $^5D_4$-$^7F_6$ transition and 540 nm for $^5D_4$-$^7F_5$), the emission can thus be collected in a single spectrogram with a fine spectral resolution. For best accuracy, it requires measuring the degree of polarization (DOP) to select the transition having highest DOP. Note that DOP can be improved even for the same transition when the crystal structure of host matrix is optimized. For example, in our previous study, the DOP was found to be much higher for monazite $LaPO_4$:Eu nanocrystal than rhabdophane $LaPO_4$:Eu nanocrystal[40]. When considering the use of Er^{3+}, although it exhibits a transition from both MD and ED, the MD emission appears only in the infrared range, away from the emission of ED. If the emissions are not adjacent in wavelength, it would be difficult to see both emissions on one spectrogram with good resolution. In this case, one can choose either ED or MD emission, showing the highest DOP to perform the orientation calculation. When using only one transition, it requires two spectrograms obtained from an orthogonal polarization. Finally, it should be noted that different transitions must be well-separated to take into account the different polarization nature of each.

Reference spectra normalization

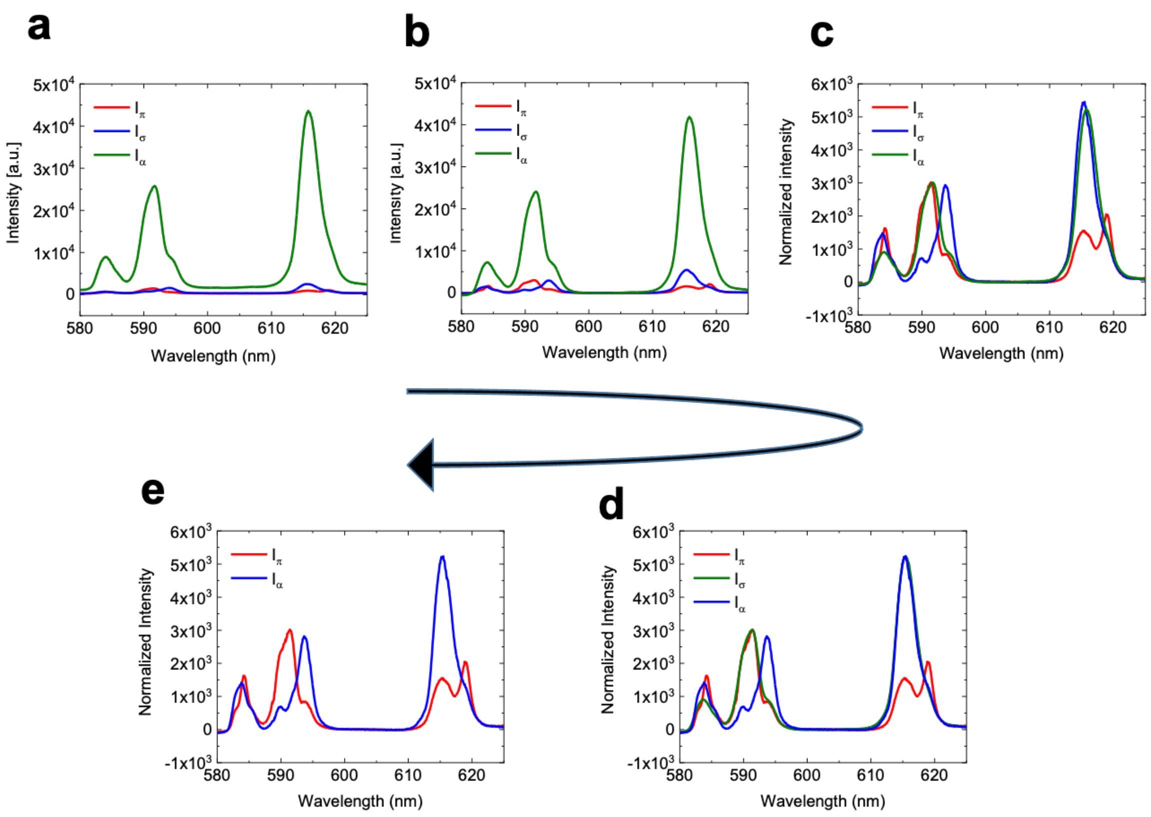

Supplementary Figure 16. Normalization process for the reference spectra. (**a**) Raw spectrum of π, σ and α signal, showing a large difference in their global intensity. (**b**) Baseline subtracted spectrum of π, σ and α signal. Average intensity value of wavelength where Eu^{3+} luminescence is not present is subtracted for each spectrum. **(c)** Normalized α signal to π signal **(d)** Normalized σ signal to α signal **(e)** Intensity corrected reference spectrum used for orientation analysis.

Polarization-dependency correction using white light source

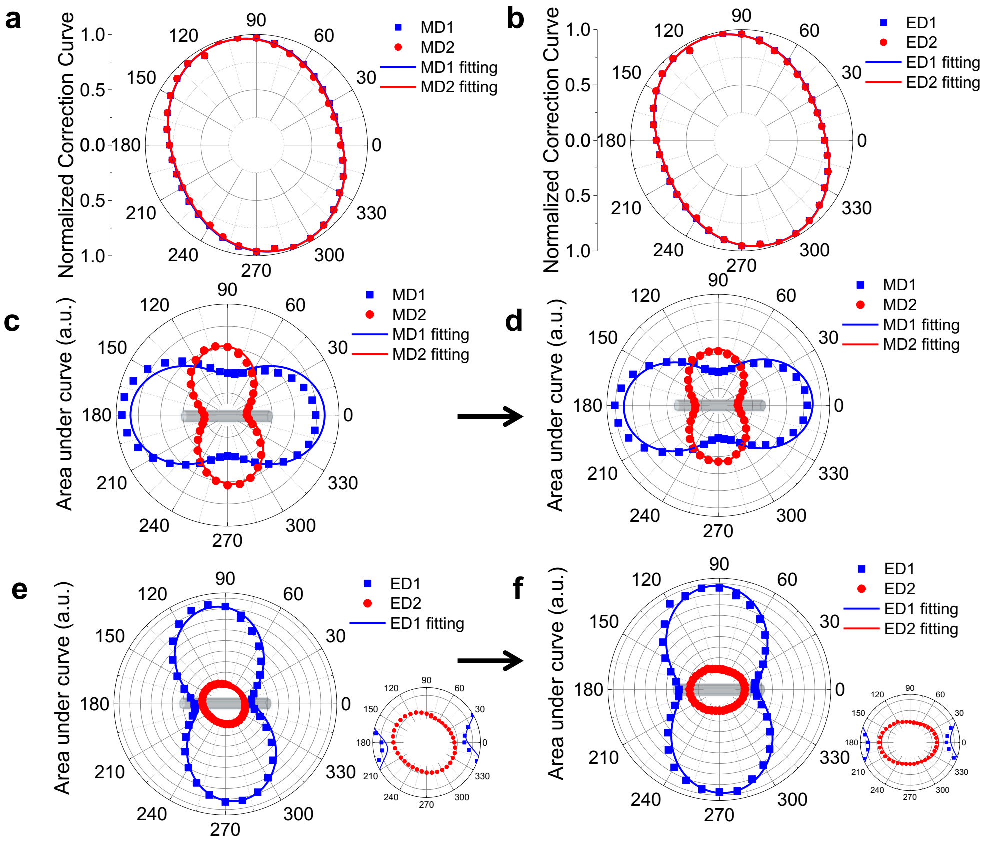

Supplementary Figure 17. **(a,b)** Polar diagram of normalized area under curve of unpolarized white light background source spectra within selected wavelength ranges (**Figure 2i)** for (a) MD1 and MD2, (b) ED1 and ED2. Polar diagrams of selected wavelength ranges for **(c,d)** MD1 and MD2, **(e,f)** ED1 and ED2 of $NaYF_4$:Eu polarized photoluminescence spectra **(c,e)** before and **(d,f)** after white light correction. Correction was made for each analyzer angle as : $I_{corrected} = I_{raw} / I_{white\text{-}source}$.

The incident ray to the dielectric surface retards when they are reflected or/and transmitted. Similarly, most of optical pass- and band- filters and mirrors hold different magnitude of transmittance and reflectance depending on the polarization state of incoming wave, which induce the detected signal different with intrinsic polarization emission. Even though the white light background source is un-polarized, the polar diagrams (**Supplementary Figure 17a,b**) observed shows obvious ellipsoid due to reason mentioned above. In an ideal case of unpolarized light, the polar diagram should show a perfect circle. This polarization dependency result in distorted polar diagram neither parallel nor orthogonal to the rod c-axis (**Supplementary Figure 17c**,**e**). By applying white light background as a compensator ($I_{corrected} = I_{raw} / I_{white\text{-}source}$), intrinsic polarization behavior can be obtained (**Supplementary Figure 17d,f**).

Supplementary References

[1] Kim, J. et al. Monitoring the orientation of rare-earth-doped nanorods for flow shear tomography. *Nature Nanotechnology* **12**, 914 (2017).

[2] Rodríguez-Sevilla, P. et al. Determining the 3D orientation of optically trapped upconverting nanorods by in situ single-particle polarized spectroscopy. *Nanoscale* **8**, 300-308 (2016).

[3] Green, K. K., Wirth, J. & Lim, S. F. Nanoplasmonic upconverting nanoparticles as orientation sensors for single particle microscopy. *Scientific Reports* **7**, 762 (2017).

[4] Ohmachi, M. et al. Fluorescence microscopy for simultaneous observation of 3D orientation and movement and its application to quantum rod-tagged myosin V. *Proceedings of the National Academy of Sciences* **109**, 5294-5298 (2012).

[5] Rosenberg, S. A., Quinlan, M. E., Forkey, J. N. & Goldman, Y. E. Rotational Motions of Macro- molecules by Single-Molecule Fluorescence Microscopy. *Accounts of Chemical Research* **38**, 583-593 (2005).

[6] Sosa, H., Peterman, E. J. G., Moerner, W. E. & Goldstein, L. S. B. ADP-induced rocking of the kinesin motor domain revealed by single-molecule fluorescence polarization microscopy. *Nature Structural Biology* **8**, 540-544 (2001).

[7] Adachi, K. et al. Stepping rotation of F1-ATPase visualized through angle-resolved single-fluorophore imaging. *Proceedings of the National Academy of Sciences* **97**, 7243-7247 (2000).

[8] Ha, T., Laurence, T. A., Chemla, D. S. & Weiss, S. Polarization spectroscopy of single fluorescent molecules. *The Journal of Physical Chemistry B* **103**, 6839-6850 (1999).

[9] Backer, A. S., Lee, M. Y. & Moerner, W. E. Enhanced DNA imaging using super-resolution microscopy and simultaneous single-molecule orientation measurements. *Optica* **3**, 659-666 (2016).

[10] Valades Cruz, C. A. et al. Quantitative nanoscale imaging of orientational order in biological filaments by polarized superresolution microscopy. *Proceedings of the National Academy of Sciences* **113**, E820-E828 (2016).

[11] Yamamoto, J. et al. Rotational diffusion measurements using polarization-dependent fluorescence correlation spectroscopy based on superconducting nanowire single-photon detector. *Opt. Express* **23**, 32633-32642 (2015).

[12] Tsay, J. M., Doose, S. & Weiss, S. Rotational and translational diffusion of peptide-coated CdSe/CdS/ZnS nanorods studied by fluorescence correlation spectroscopy. *Journal of the American Chemical Society* **128**, 1639-1647 (2006).

[13] Sezgin, E. et al. Measuring nanoscale diffusion dynamics in cellular membranes with super-resolution STED–FCS. *Nature Protocols* **14**, 1054-1083 (2019).

[14] Lethiec, C. et al. Measurement of three-dimensional dipole orientation of a single fluorescent nanoemitter by emission polarization analysis. *Physical Review X* **4**, 021037 (2014).

[15] Early, K. T. et al. Linear dipole behavior in single CdSe-Oligo(phenylene vinylene) nanostructures. *ACS Nano* **3**, 453-461 (2009).

[16] Chung, I., Shimizu, K. T. & Bawendi, M. G. Room temperature measurements of the 3D orientation of single CdSe quantum dots using polarization microscopy. *Proceedings of the National Academy of Sciences* **100**, 405-408 (2003).

[17] Empedocles, S. A., Neuhauser, R. & Bawendi, M. G. Three-dimensional orientation measurements of symmetric single chromophores using polarization microscopy. *Nature* **399**, 126-130 (1999).

[18] Forkey, J. N., Quinlan, M. E., Alexander Shaw, M., Corrie, J. E. T. & Goldman, Y. E. Three-dimensional structural dynamics of myosin V by single-molecule fluorescence polarization. *Nature* **422**, 399-404 (2003).

[19] Toprak, E. et al. Defocused orientation and position imaging (DOPI) of myosin V. *Proceedings of the National Academy of Sciences* **103**, 6495-6499 (2006).

[20] Dedecker, P. et al. Defocused Wide-field Imaging Unravels Structural and Temporal Heterogeneity in Complex Systems. *Advanced Materials* **21**, 1079-1090 (2009).

[21] Lieb, M. A., Zavislan, J. M. & Novotny, L. Single-molecule orientations determined by direct emission pattern imaging. *J. Opt. Soc. Am. B* **21**, 1210-1215 (2004).

[22] Patra, D., Gregor, I. & Enderlein, J. Image analysis of defocused single-molecule images for three-dimensional molecule orientation studies. *The Journal of Physical Chemistry A* **108**, 6836-6841 (2004).

[23] Li, T. et al. Three-dimensional orientation sensors by defocused imaging of gold nanorods through an ordinary wide-field microscope. *ACS Nano* **6**, 1268-1277 (2012).

[24] Lethiec, C. et al. Polarimetry-based analysis of dipolar transitions of single colloidal CdSe/CdS dot-in-rods. *New Journal of Physics* **16**, 093014 (2014).

[25] Böhmer, M. & Enderlein, J. Orientation imaging of single molecules by wide-field epifluorescence microscopy. *J. Opt. Soc. Am. B* **20**, 554-559 (2003).

[26] Brasselet, S. et al. In Situ Diagnostics of the Crystalline Nature of Single Organic Nanocrystals by Nonlinear Microscopy. *Physical Review Letters* **92**, 207401 (2004).

[27] Mayer, L. et al. Single KTP nanocrystals as second-harmonic generation biolabels in cortical neurons. *Nanoscale* **5**, 8466-8471 (2013).

[28] Winters, D. G., Smith, D. R., Schlup, P. & Bartels, R. A. Measurement of orientation and susceptibility ratios using a polarization-resolved second-harmonic generation holographic microscope. *Biomed. Opt. Express* **3**, 2004-2011 (2012).

[29] Tiaho, F., Recher, G. & Rouède, D. Estimation of helical angles of myosin and collagen by second harmonic generation imaging microscopy. *Opt. Express* **15**, 12286-12295 (2007).

[30] Stoller, P., Reiser, K. M., Celliers, P. M. & Rubenchik, A. M. Polarization-modulated second harmonic generation in collagen. *Biophysical Journal* **82**, 3330-3342 (2002).

[31] Xiao, L. & Yeung, E. S. Optical Imaging of Individual Plasmonic Nanoparticles in Biological Samples. *Annual Review of Analytical Chemistry* **7**, 89-111 (2014).

[32] Chang, W.-S., Ha, J. W., Slaughter, L. S. & Link, S. Plasmonic nanorod absorbers as orientation sensors. *Proceedings of the National Academy of Sciences* **107**, 2781-2786 (2010).

[33] Ha, J. W., Marchuk, K. & Fang, N. Focused Orientation and Position Imaging (FOPI) of Single Anisotropic Plasmonic Nanoparticles by Total Internal Reflection Scattering Microscopy. *Nano Letters* **12**, 4282-4288 (2012).

[34] Xiao, L., Ha, J. W., Wei, L., Wang, G. & Fang, N. Determining the Full Three-Dimensional Orientation of Single Anisotropic Nanoparticles by Differential Interference Contrast Microscopy. *Angewandte Chemie International Edition* **51**, 7734-7738 (2012).

[35] Ha, J. W., Sun, W., Stender, A. S. & Fang, N. Dual-Wavelength Detection of Rotational Diffusion of Single Anisotropic Nanocarriers on Live Cell Membranes. *The Journal of Physical Chemistry C* **116**, 2766-2771 (2012).

[36] Marchuk, K., Ha, J. W. & Fang, N. Three-Dimensional High-Resolution Rotational Tracking with Superlocalization Reveals Conformations of Surface-Bound Anisotropic Nanoparticles. *Nano Letters* **13**, 1245-1250 (2013).

[37] Wang, G., Sun, W., Luo, Y. & Fang, N. Resolving Rotational Motions of Nano-objects in Engineered Environments and Live Cells with Gold Nanorods and Differential Interference Contrast Microscopy. *Journal of the American Chemical Society* **132**, 16417-16422 (2010).

[38] Xiao, L., Qiao, Y., He, Y. & Yeung, E. S. Imaging Translational and Rotational Diffusion of Single Anisotropic Nanoparticles with Planar Illumination Microscopy. *Journal of the American Chemical Society* **133**, 10638-10645 (2011).

[39] Wackenhut, F., Failla, A. V., Züchner, T., Steiner, M. & Meixner, A. J. Three-dimensional photoluminescence mapping and emission anisotropy of single gold nanorods. *Applied Physics Letters* **100**, 263102 (2012).

[40] Chaudan, E. et al. Polarized Luminescence of anisotropic LaPO4:Eu nanocrystal polymorphs. *Journal of the American Chemical Society* **140**, 9512-9517 (2018).

[41] Bünzli, J.-C. G. & Eliseeva, S. V. in *Lanthanide Luminescence: Photophysical, Analytical and Biological Aspects* (eds Pekka Hänninen & Harri Härmä) 1-45 (Springer Berlin Heidelberg, 2011).
