# Supplementary material for: Measuring 3D orientation of nanocrystals via polarized luminescence of rare-earth dopants
Source: Nat Commun. 2021 Mar 29;12:1943. doi: 10.1038/s41467-021-22158-4 (PMC8007814; doi:10.1038/s41467-021-22158-4)
Supplement: Supplementary file 2 — Description of Additional Supplementary Files [file 41467_2021_22158_MOESM2_ESM.pdf]

Description of Additional Supplementary Files

File Name: Supplementary Data 1
Description: **Full dataset of analyzed orientation of randomly selected $NaYF_4$:Eu nanorods.** All the calculated/measured values are rounded up. "N/A" indicates nanorods that in-plane angle φ' was not obvious to measure from CCD captured image due to very low ellipticity (oriented close to vertical to the substrate or smaller in size than the resolution of CCD camera). "Error" indicates nanorods that calculated $\cos^2\theta$ or/and $\sin^2\varphi$ is out of sinusoidal function range (0-1), thus conversion to (θ', φ') could not be made. The origin of the error is discussed in the main text (Section 2.2.3). **Table SD3** shows the statistics of analyzed orientation pre presented in **Table SD1,2**. Nanorods shown as "N/A" or "Error" is not included in error calculation.

Table SD1. Rods oreinted in PVA film (3D sample). Calculated values of the trigonometric functions of (θ, φ) and the absolute values of (θ', φ') of $NaYF_4$:Eu nanorods randomly oriented 3D in polymer film. Orientation is analyzed using spectral fitting analysis and area under curve ratiometry (AUCR). Calculated in-plane angle φ' is compared with measured φ' on the elliptical shape of CCD captured nanorod image (data not shown). Out-of-plane angle (θ') remains incomparable. Results shown here is denoted as "3D sample" in **Table SD3**.

Table SD2. Rods lying on the substrate (2D sample). Calculated values of the trigonometric functions of (θ, φ) and the absolute values of (θ', φ') of $NaYF_4$:Eu nanorods lying on the substrate. Orientation is analyzed using spectral fitting analysis and area under curve ratiometry (AUCR). Calculated in-plane angle φ' is compared with measured φ' on the elliptical shape of CCD captured nanorod image (data not shown). Calculate out-of-plane angle θ' is compared with known $\theta' = 90^o$. Results shown here is denoted as "2D sample" in **Table SD3**.

Table SD3. Statistics of analyzed orientation presented in Table SD1,2.
